# Reduction of inflammatory biomarkers underlies extracellular vesicle mediated functional recovery in an aged monkey model of cortical injury

**DOI:** 10.3389/fnagi.2025.1605144

**Published:** 2025-07-09

**Authors:** Ryan P. McCann, Bethany Bowley, Monica Pessina, Qiong Yang, Hongqi Xin, Sarah A. DeVries, Mingjin Wang, Yi Zhang, Michael Chopp, Zhenggang Zhang, Douglas L. Rosene, Ella Zeldich, Maria Medalla, Tara L. Moore

**Affiliations:** ^1^Graduate Program for Neuroscience, Boston University, Boston, MA, United States; ^2^Department of Anatomy and Neurobiology, Boston University Chobanian and Avedisian School of Medicine, Boston, MA, United States; ^3^Department of Biostatistics, Boston University School of Public Health, Boston, MA, United States; ^4^Department of Neurology, Henry Ford Health, Detroit, MI, United States; ^5^Center for Systems Neuroscience, Boston University, Boston, MA, United States

**Keywords:** rhesus monkey, extracellular vesicle, cortical injury, microglia, inflammation, motor function

## Abstract

Cortical injury results in inflammation and cell death that can cause disability, especially in the aged population. Previous studies from our group have demonstrated the efficacy of bone marrow mesenchymal stromal cell derived extracellular vesicles (MSC-EVs) as a therapeutic to mitigate damage and enhance recovery in our aged monkey model of cortical injury. In the first 3–5 weeks following injury to the hand representation of the primary motor cortex, monkeys treated intravenously with MSC-EVs exhibited a more rapid and complete recovery of fine motor grasp compared to vehicle-treated monkeys. However, whether recovery and treatment are associated with temporal changes in peripheral or central biomarkers of inflammation remain unknown. The current study used the highly sensitive Olink^®^ Proximity Extension Assay to assess inflammatory protein biomarkers in blood and CSF across a 6-week recovery period in aged female monkeys. MSC-EV treatment promoted a sustained downregulation of pro-inflammatory proteins in plasma across the entire recovery period, and a transient downregulation of anti-inflammatory proteins at 2 weeks post-injury. Functional annotation and pathway analyses showed that the plasma proteins downregulated with MSC-EV treatment were associated with the suppression of pro-inflammatory signaling. Further, immunolabeling of perilesional brain tissue harvested 6-weeks post injury showed an increase in homeostatic microglial phenotypes with MSC-EV treatment. Downregulation of inflammatory markers in plasma and brain tissue were positively correlated with improved functional recovery. These data suggest that MSC-EVs facilitate recovery of function after brain injury, in part, via sustained suppression of both peripheral and central pro-inflammatory signaling across recovery.

## 1 Introduction

Cortical injury due to trauma, stroke, or other insults, is a leading cause of long-term disability in the aged population. Brain injury, even mild cases, can result in long-term impairments in cognition, motor function and affect, which impact activities of daily living and overall well-being (Hoge et al., 2008). While some degree of recovery after injury may occur with the development of compensatory function, full recovery is rare (Grefkes and Ward, 2014).

The inflammatory response caused by cortical injury, which is regulated by mediators that include cytokines, chemokines, and growth factors, can modulate both damage or repair depending on the stage of recovery (Barone and Feuerstein, 1999). The acute phase of recovery after injury involves a rapid pro-inflammatory response necessary to recruit central and peripheral immune cells to the injured area to contain and clear the damage (Kim et al., 2014). Following the pro-inflammatory response, an anti-inflammatory repair phase facilitates plasticity and reorganization of the surviving neuronal circuits to regain function. However, excessive and prolonged chronic inflammation can lead to secondary damage and reduced plasticity, thereby hindering functional recovery (Schimmel et al., 2017; Galgano et al., 2017). There are currently no FDA-approved therapies to alter the chronic inflammatory environment and facilitate full recovery of function following injury. However, recent studies have explored the post-injury inflammatory response and secondary damage cascade as promising therapeutic targets (Ting et al., 2020; Lambertsen et al., 2019).

One potential therapeutic intervention that has shown efficacy in multiple models of brain injury and neurodegenerative diseases is mesenchymal stromal-cell derived extracellular vesicles (MSC-EVs) (Hao et al., 2022; Xin et al., 2013a; Xin et al., 2013b; Xin et al., 2021). Bone marrow MSCs are multipotent cells with immunosuppressive and immunoprivileged properties (Keating, 2006), that are thought to be effected by the EVs they release. The EVs released by MSCs are lipid bound nanovesicles carrying proteins, lipids, and microRNA cargo that have many beneficial anti-inflammatory, neuroregenerative, and neuroprotective effects (Zhang K. et al., 2022). Studies from our group have shown that intravenous administration of MSC-EVs at 24 h and again 2 weeks following injury facilitates recovery of fine motor hand function in aged female rhesus monkeys (Moore et al., 2019; Moore et al., 2016; Moore et al., 2013). We have developed a cortical injury model in rhesus monkeys, in which a lesion to the hand representation of the primary motor cortex (M1) causes grasp impairment. The monkeys that were treated with MSC-EVs showed a significantly greater extent and more rapid recovery of fine motor grasp function in the first 3–5 weeks following injury (Moore et al., 2019). Post-mortem analyses of brain tissue harvested 16 weeks following injury revealed that MSC-EVs reduced injury-related microglial activation (Go et al., 2020), neuronal damage (Medalla et al., 2020) and promoted neuroplasticity and reorganization in perilesional M1 and premotor cortices (Go et al., 2021; Calderazzo et al., 2022; Zhou et al., 2023). However, the time course and mechanism of MSC-EV action remains unclear in our model. Further, since MSC-EVs were administered intravenously, it is important to assess if and how treatment affects the temporal changes and relationships between peripheral and central biomarkers across recovery.

Therefore, the current study used a new cohort of monkeys which were assessed across a 6-week post injury recovery period. Plasma and cerebrospinal fluid (CSF) were collected across recovery from this cohort and analyzed for inflammatory proteins using the Olink® Proximity Extension Assay (PEA), a novel high sensitivity multiplex protein assay. Brain tissue was then harvested at 6 weeks post-injury and analyzed for the expression of inflammatory microglial phenotypes, identified by morphology and expression of major histocompatibility complex II (MHC-II), a marker for immune-activation (Lue et al., 2010). We compared these new findings in brain tissue at 6-weeks post injury, with our previous data showing MSC-EV related brain changes at 14–16-weeks post-injury in age matched female monkeys (Go et al., 2020). Collectively, these data provide evidence that MSC-EV treatment can lead to a sustained suppression of plasma inflammatory biomarkers across acute and chronic stages of recovery, and reduction of microglial inflammation at 6-weeks post-injury, supporting the recovery of fine motor function.

## 2 Methods

### 2.1 Subjects

**Figure 1:**
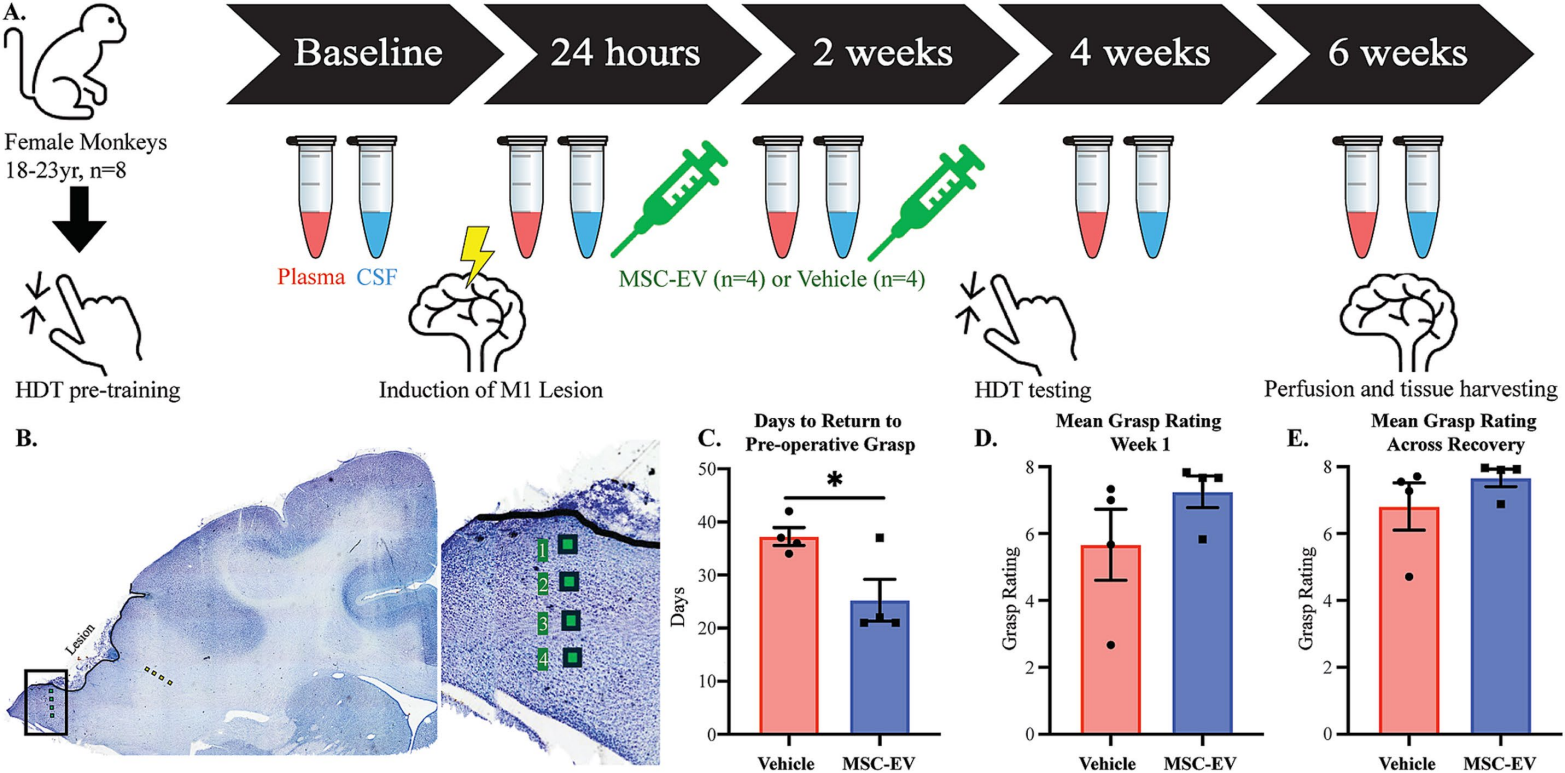
Schematic of experimental design and assessments of functional recovery after cortical injury. **(A)** Timeline of the experimental design and model. Monkeys began with pre-training; upon completion they had surgery to induce a lesion to the hand representation of the motor cortex. Treatment was administered at 24 h and 2 weeks to both MSC-EV treated monkeys (*n* = 4) and vehicle control monkeys (*n* = 4). Plasma and CSF were collected at baseline, 24 h, 2 weeks, 4 weeks and 6-weeks post-injury. Post-injury testing began 15 days following lesion surgery. Perfusion and tissue harvest for 8 monkeys occurred 6 weeks following surgery. **(B)** Nissl-stained coronal section through the lesion in M1 showing sites sampled and imaged for IHC analyses of microglia in perilesional gray (green squares) and sublesional white (yellow squares) matter. **(C–E)** Behavioral outcome measures of motor recovery: **(C)** The number of days it takes to return to pre-operative grasp. All monkeys that did not recover within 6 weeks are assigned a 42 for analysis purposes. **(D)** The mean grasp rating in the first week of post-injury testing (days 15–21 following cortical injury). **(E)** The mean grasp rating across the post-injury testing period (days 15–42). **p < 0.01, *p < 0.05. Eppendorf tube adapted from 10.5281/zenodo.6808872.

Eight aged female rhesus monkeys (*Macaca mulatta*) (18–23 years, equivalent to approximately 54–69-year-old humans) were used in this study and were randomly assigned to MSC-EV treated versus vehicle treated groups (*n* = 4 for each group). The experimental timeline was based on our previous work (Moore et al., 2019) and is summarized in Figure 1A. Briefly, the monkeys were trained for 4 weeks on a task of fine motor function of the hand prior to surgical induction of a lesion in the hand representation of the primary motor cortex (M1). Monkeys were administered either MSC-EVs or vehicle intravenously at 24 h and then again 2 weeks after surgery and then began retesting on the same fine motor task for 4 weeks. Cerebrospinal fluid (CSF) and blood (for plasma) were drawn at multiple time points across the recovery period, and monkeys were then euthanized at 6 weeks post injury for harvesting of brain tissue (Figure 1B). Data from brain tissue were also compared to our archived data from our previous studies (Moore et al., 2019; Go et al., 2020; Zhou et al., 2023) with a cohort of aged female monkeys (16–26 years) from which brains were harvested 16-weeks post injury (MSC-EV *n* = 5, Vehicle *n* = 5).

All monkeys were obtained from national primate research facilities or private vendors and had known birth dates and complete health records. Only female monkeys were available at the time of this study. Monkeys received medical examinations and magnetic resonance imaging to ensure there were no occult health problems or neurological damage. Monkeys were housed in the Animal Science Center of Boston University Chobanian and Avedisian School of Medicine which is AAALAC accredited. All procedures were approved by the Boston University Institutional Animal Care and Use Committee (2018–00053).

### 2.2 Pre-operative training on fine motor function task

Testing of the fine motor function of the hand is fully described in Moore et al. (2019). In brief, monkeys were trained on a task of fine motor function, the Hand Dexterity Task (HDT), using a testing apparatus that quantifies the response latency and video records responses from each hand (Pessina et al., 2019). The HDT is a modified version of a Kluver board (Klüver, 1935) and requires precise control of the digits, particularly opposition of the thumb and index finger, to retrieve a small, visible food reward from two different size round wells in a plexiglas tray.

### 2.3 Electrophysiological mapping of the hand representation in primary motor cortex

All surgical procedures are described in detail in Moore et al. (2019) and briefly summarized as follows: To create reproducible cortical injury and motor deficits, the precentral gyrus was systematically mapped using electrical stimulation delivered through a small monopolar silver ball electrode placed on the surface of the pia to evoke movements. During each stimulation, a trained observer noted muscle movements (e.g., distinct movement or twitches of muscle) in specific areas of the hand, forearm or arm, both visually and by palpation. Responses were recorded on a calibrated photograph creating a cortical surface map of the hand area that was used to guide placement of the lesion.

### 2.4 Induction of selective cortical injury in primary motor cortex hand area

Using the map described above, the cortical injury was induced by inserting a small glass suction pipette under the pia to bluntly transect the small penetrating arterioles as they enter the underlying cortex. Since the hand representation is known to extend down the rostral bank of the central sulcus, the sulcus was opened down to the fundus along the length of the gyral hand representation by microdissection with a small glass pipette and the pia was dissected with the glass pipette all the way down to the fundus of the sulcus.

### 2.5 MSC-EV preparation

Bone marrow was harvested from the head of the humerus of a young female monkey (~5 years of age) in our colony, as described in detail in Moore et al. (2019). Briefly, the monkey was sedated with ketamine (10 mg/kg, IM) and anesthetized with sodium pentobarbital (15–25 mg/kg IV). After sterilizing the field, bone marrow was aspirated from the humerus using a bone biopsy needle. After biopsy, monkeys were treated with buprenex (0.01–0.03 mg/kg). The collected bone marrow was shipped on ice, the same day to the Henry Ford Health. Upon arrival, the bone marrow sample was centrifuged (4,000xg for 15 min) to separate cells. The MSCs were isolated and cultured. MSC-EVs were extracted from the culture media, and purified via multi-step centrifugation and filtration, as described (Moore et al., 2019). The size distribution and concentration of the MSC-EVs was measured by qNano (Izon, Cambridge, MA), which confirmed a distribution of EVs ranging from 60 to 300 nm, with a prominent peak observed at 157 nm. Expression of EV markers (CD9, CD63) were confirmed via western blot (Supplementary Figure 1; Théry et al., 2018; Welsh et al., 2024). Each treated monkey received 4 × 10^11^ particles/kg in 10 mL of PBS and control monkeys received 10 mL of PBS. Both MSC-EV and Vehicle doses were administered intravenously 24 h and again 2 weeks after cortical injury (Figure 1A). Dosing concentration and intervals were identical to our previous work in Moore et al. (2019), which were based on studies in rodents (Xin et al., 2013a; Xin et al., 2013b).

### 2.6 Post-operative motor testing

Post-operative testing on the HDT began 2 weeks after surgery and continued for 4 weeks (Figure 1A). Testing occurred three times per week following injury to mimic rehabilitation measures used in human stroke patients.

### 2.7 Grasp pattern assessment

We developed a Non-Human Primate Grasp Assessment Scale (GRAS) (Moore et al., 2016; Pessina et al., 2019) to detect and quantify significant impairments in fine motor function of the hand and to document recovery of function of individual digits and the precise finger-thumb pinch used by monkeys to retrieve food morsels. Using the video-recorded responses, this scale allows us to distinguish between compensatory grasp patterns and a return to pre-injury grasp patterns. A maximum score of 8 is normal grasp function (Pessina et al., 2019).

### 2.8 Blood and CSF draws

Cerebrospinal fluid (CSF) and blood (for plasma) were drawn at multiple time points across the study period (Pre-surgery/baseline, 24 h, 2 weeks, 4 weeks, and at the terminal timepoint, approximately 6 weeks post-injury, Figure 1A). At the timepoints that coincide with MSC-EV or vehicle administration (24 h, 2 weeks), blood and CSF were collected immediately prior to treatment administration. Briefly, monkeys were sedated with Ketamine (10 mg/kg IM), CSF was drawn from the cisterna magna and transferred into a K3 EDTA tube and blood was drawn from the femoral vein into a K3 EDTA tube with a Vacutube and both were immediately placed in 4°C for temporary storage (less than 2 h). Plasma was centrifuged at 3,000 RPM for 15 min at 4°C and then stored in 250 μL aliquots at −80°C. CSF was centrifuged at 6,000 RPM for 1 min and stored in 100 μL aliquots and frozen at −80°C until processing.

### 2.9 Perfusion and brain tissue harvesting

Following post-operative motor testing, at 6-weeks post-injury (Figure 1A), brain tissue was harvested in a terminal procedure as follows: Monkeys were sedated with Ketamine (10 mg/kg IM) then anesthetized with either sodium pentobarbital (25 mg/kg IV to effect) or propofol (2.5 mg/kg) followed by exsanguination during transcardial perfusion of the brain. The perfusion began with cold Krebs-Heinsleit buffer (4°C, pH 7.4) during which, small tissue biopsies from pre-motor cortex were collected for separate studies, and perfusate was switched to 8 L of 4% paraformaldehyde (30°C, pH 7.4) to fix the remainder of the brain. Brains were blocked *in situ*, in the coronal plane, removed from the skull, and drop fixed with 4% paraformaldehyde for 24 h (4°C, pH 7.4), then cryoprotected in a solution of 0.1 M phosphate buffer, 10% glycerol, and 2% DMSO followed by buffer with 2% DMSO and 20% glycerol. Brains were then flash-frozen in −75°C 2-methylbutane and stored at −80°C until cut on a microtome in the coronal plane into series (8 series of 30-μm sections, and one 60-μm section series). Sections were stored at −80°C in a cryoprotectant of 15% glycerol in buffer (Estrada et al., 2017).

### 2.10 Olink^®^ proximity extension assay

To assess levels of inflammatory proteins in plasma and CSF, the Olink^®^ PEA Inflammation panel (Olink, Uppsala, Sweden) was used on the samples collected across recovery. Samples from baseline, 24 h, 2 weeks, 4 weeks, and 6 weeks were sent to the Olink^®^ analysis facility in Waltham, MA. This multiplex protein measurement assay (92 inflammatory proteins) used two antibodies for every protein of interest. Each antibody was tagged with a single stranded DNA fragment. When two paired antibodies properly bind the targeted protein of interest, the DNA fragments hybridized. The hybridized DNA fragments were extended to create barcoded DNA fragments that were specific to the target protein. The number of coded fragments in the DNA library was specific to the relative concentration of the proteins (Assarsson et al., 2014). Positive control samples were run to determine efficacy and negative control samples were run to determine the limit of detection (Supplementary Table S1). Multiplex qPCR was used to quantify the levels of target proteins, and a normalized protein concentration (NPX) was reported in log2 format. For this study, all NPX log2 values were converted to base values and normalized to baseline protein measurements from their own baseline sample. Proteins that did not reach our criterion (more than 25% of samples did not reach detectable levels, or any monkey baseline values did not reach detectable levels) were excluded from analyses so that in plasma, 64 of 92 proteins reached criterion, and in CSF 39 of 92 proteins reached criterion (Supplementary Tables S2, S9). A similar rate of protein detection with the Olink^®^ PEA was reported for the CSF from a Rhesus monkey model of SARS-Cov-2 infection (Verma et al., 2021). We then further analyzed 36 of these proteins in plasma and 23 in CSF that are known to be implicated in stroke and cortical injury by normalizing the values against baseline and comparing across treatment groups and timepoints (Supplementary Tables S4, S11).

### 2.11 Immunofluorescence and confocal imaging

Immunofluorescence was performed on 30 μm brain tissue coronal sections stored at −80°C from the same cohort used in the biomarker analysis (4 MSC-EV and 4 vehicle), based on our previous work (Go et al., 2020; Zhou et al., 2023). Serial coronal sections were selected (*n* = 2 sections per case) at the level of the lesion in M1. Free-floating sections were washed in 0.01 M PBS (3 × 10 min) and then incubated in 50 mM glycine for 1 h (at 25°C, room temperature, while rocking). Sections were washed (3×10 min, PBS) and then incubated in 10 mM sodium citrate buffer (pH = 8.5) for 20 min in water bath at 75°C for antigen retrieval. Sections were washed then placed in pre-block solution (5% Bovine Serum Albumin, 5% Normal Donkey Serum, 0.2% Triton-X in 0.01 M Phosphate buffered saline) for 1 h (at 25°C, room temperature, while rocking). All antibodies were diluted in an antibody diluent solution containing 0.2% acetylated Bovine Serum Albumin, 1% Normal Donkey Serum, 0.1% Triton-X in 0.1 M Phosphate buffer. Sections were incubated in primary antibody against Iba1 and P2RY12 for enhancement of microglial processes (1:500 Rabbit anti-Iba1, Wako Cat# 019–19741; 1:250 Rabbit anti-P2RY12, Abcam Cat# ab1030066), MHCII (1:100 Mouse anti-LN3, MPBiosystems, Cat# 08634801), first in a variable wattage microwave (Biowave, Ted Pella, Inc., Redding, CA, United States) for 20 min (at 150 Watts and 25°C), then followed by an incubation at 4°C (while rocking) for approximately 65 h. Sections were washed (3× 10 min PBS), then incubated in secondary antibodies, Donkey anti-Mouse IgG conjugated to Alexa 568 fluorescence probe (diluted 1:200, Jackson ImmunoResearch, Cat# 703–605-155) and biotinylated donkey anti-rabbit (diluted 1:200, Jackson ImmunoResearch, Cat#711–065-152), first in the microwave (Biowave) for 20 min (at 150 Watts and 25°C), followed by incubation at 4°C (while rocking) for 20 h. For 4 h at room temperature, slices were incubated in streptavidin conjugated with Alexa 405 (diluted 1:200, Invitrogen Cat# S32351). Sections were then washed (3× 10 min in 0.1 M PB) and mounted on gelatin subbed slides. Slides were coverslipped with Prolong Gold anti-fade (Invitrogen, Cat# P36930), cured in the dark for 48 h at room temperature, and then stored at 4°C, until imaged.

Sections were imaged on a Zeiss LSM 710 confocal microscope (Zeiss, Jena, Germany) at four wavelengths (405 nm, 488 nm, 561 nm, and 633 nm), using a 40x oil immersion lens (Zeiss EC Plan-Neofluar 40x/1.3 Oil DIC) at a voxel resolution of 0.21 × 0.21 × 0.5 μm. For imaging the perilesional gray matter, the surface of the lesion was identified. A total of 4 images were acquired for each region of interest in the lesion section as follows: The first image was acquired 200 μm away from the lesion surface towards the white matter. Subsequent images throughout the grey matter depth were then acquired every 400 μm, perpendicular to the lesion surface. For the white matter, the first image was acquired 200 μm away from the gray/white interface below the lesion core. Subsequent images throughout the white matter depth were then acquired every 400 μm, perpendicular to the lesion surface (Figure 1B).

All images were processed and analyzed as described in our previous studies (Go et al., 2020; Zhou et al., 2023; Medalla and Luebke, 2015). First, confocal image stacks were deconvolved using AutoQuantX3 software (MediaCybernetics, Bethesda, MD, United States) to enhance signal-to-noise ratio. Images were assessed for microglia cell counts using Neurolucida software (MBF Bioscience, Williston, VT, United States), employing stereological 3D cell counting methods. Microglia were first identified with staining for Iba1 and then categorized into the following groups according to morphology and expression (double labeling) of MHCII: Ramified/MHCII-, Ramified/MHCII+, Hypertrophic/Ameboid/MHCII-, and Hypertrophic/Ameboid/MHCII+, as previously performed in Go et al. (2020). Microglia were classified as “ramified” versus “hypertrophic/ameboid” morphology, based on criteria described in Karperien et al. (2013). Briefly, ramified morphology is characterized by a small circular soma (about 5–10 μm in diameter), and long thin processes that extend to about 2-4x the diameter of the soma. Hypertrophic morphology is characterized by an elliptical, elongated or irregular shaped soma with large aspect ratio and/or major diameters (about > 10 μm), and thick primary processes. Amoeboid morphology is characterized by large soma that either lack processes or have thick and short processes (with widths >0.5x the soma diameter and lengths about < 1x the diameter of the soma) (See results). Microglia were counted in each z-stack using 3D counting rules with inclusion and exclusion borders, and values were expressed as density (number of cells/total volume of tissue per field or area) or proportion (total number of cells counted in each type/ total cells counted per field or area), as described previously (Tsolias and Medalla, 2021; Tsolias et al., 2024; Go et al., 2020; Zhou et al., 2023).

### 2.12 Statistics

#### 2.12.1 Statistical comparisons and correlation analyses

For between-group comparisons (MSC-EV vs. vehicle) of motor recovery after injury, Student’s t-tests were performed on the measures of days to return to pre-operative grasp, and a Mann–Whitney test was performed on the mean grasp ratings in the first week of recovery and across the entire recovery period, using GraphPad Prism 10.

For analyses of plasma and CSF biomarkers, RStudio (2023) was used to perform statistical tests between-groups and between-timepoints. The full data set from Olink^®^ PEA consisted of 64/92 proteins in plasma and 39/92 proteins in CSF that reached criterion and were compared between groups within each timepoint. The Olink^®^ PEA results were reported in NPX format, which is log2, for each marker. We first assessed whether there were between-timepoint differences in raw NPX expression values, using a two-way ANOVA (treatment x timepoint) with Fisher’s LSD or Student’s *t*-test post-hoc tests for pairwise comparison in each timepoint by group (Table 1, Supplementary Table S3). To further assess the effects of treatment after injury, we then focused on between-group comparisons within each post-injury and treatment timepoint (2, 4 and 6 weeks) of 36 specific proteins of interest in plasma (Supplementary Tables S4, S5A,B) and 23 specific proteins of interest in the CSF (Supplementary Tables S11, S12), that are implicated in stroke and cortical injury as determined through literature review (Table 2). For each protein, we calculated the expression ratio from the raw NPX (2^NPX) values, and normalized each post-injury/treatment time point expression value to the baseline time point value for each individual monkey. Olink^®^ PEA results for inflammatory proteins of interest in cortical injury were analyzed using the OlinkAnalyze R package to assess longitudinal changes with the Olink^®^ two-way repeated measures ANOVA and Tukey’s post-hoc analysis.

**Table 1:** Two way ANOVA on raw plasma values.

Main effect of timepoint	
Variables	*p* value
‘VEGFα’	7E-07
‘LAPTGFβ’	0.011
‘uPA’	0.006
‘IL6’	7E-14
‘MCP1’	0.088
‘CXCL11’	2E-06
‘OSM’	0.003
‘MCP4’	7E-06
‘MMP1’	0.0005
‘IL10Rβ’	0.0388
‘PDL1’	1E-06
‘TRANCE’	1E-07
‘HGF’	0.0028
‘CXCL6’	0.047
‘MCP2’	0.0085
‘TWEAK’	2E-05
‘ADA’	0.051
‘TNFβ’	0.0004
‘CASP8’	0.034
‘CSF1’	2E-07

Post-hoc 24-h vs baseline						
Diff in both	*p* value Veh	*p* value EV	Diff in Veh	*p* value	Diff in EV	*p* value
‘VEGFα’	1.571E-06	0.0002322	‘OSM’	0.0111	‘CD6’	0.0002
‘IL6’	1.083E-10	6.28E-07	‘PDL1’	0.00037	‘MMP1’	0.0054
‘CXCL11’	0.0006075	4.556E-05	‘CASP8’	0.03451	‘IL10Rβ’	0.0078
‘MCP4’	0.0016126	8.652E-06	‘MCP1’	0.01282	‘MMP10’	0.0023
‘TNFβ’	0.0111825	0.031833	-	-	-	-
‘CSF1’	1.821E-05	1.629E-05	-	-	-	-
‘CXCL10’	0.0098878	7.239E-06	-	-	-	-

Post-hoc 2 weeks vs baseline				Post-hoc 2 weeks between group	
Diff in Veh	*p* value	Diff in Veh	*p* value	Diff EV-Veh	*p* value
‘OPG’	0.0172857	‘TNFα’	0.0385044	‘OPG’	0.0387227
‘LAPTGFβ1’	0.0062673	‘CCL3’	0.0212121	‘IL18R1’	0.0267304
‘uPA’	0.0366839	‘CXCL6’	0.0482761	‘CD5’	0.0040459
‘CCL4’	0.0342086	‘MCP2’	0.0452177	‘CCL3’	0.0242301
‘CD6’	0.0340195	‘CX3CL1’	0.0240589	‘CXCL6’	0.0097379
‘TGFα’	0.0381166	‘TWEAK’	0.0199393	‘CX3CL1’	0.0052756
‘IL18R1’	0.0459286	-	-	‘TWEAK’	0.0231395
‘HGF’	0.0022212	-	-	-	-

Group differences in both pro- and anti-inflammatory plasma biomarkers from raw NPX data. A two-way ANOVA was used to assess treatment by timepoint analysis, and a Fisher’s LSD or Student’s *t*-test post-hoc analysis was used to identify significant differences. The protein name and *p*-value from both the main effect of timepoint, and the post-hoc of 24 h vs. baseline, 2 weeks vs. baseline are presented in this table, and 2-weeks between group. The complete data set can be found in Supplementary Table S3.

**Table 2:** Group differences in normalized Olink PEA.

Biomarker	UniProt	Timepoint	Difference	Adjusted P	Significance	Cohen’s d	Reference
Pro-inflammatory							
CCL11	P51671	2 weeks	−0.317	0.00270	**	−1.735	Lieschke et al. (2019)
		4 weeks	−0.194	0.04757	*	−1.259	
		6 weeks	−0.179	0.06556		−0.967	
CCL19	Q99731	2 weeks	−0.481	0.01948	*	−1.542	Nossent et al. (2017), Che et al. (2023)
CCL20	P78556	2 weeks	−0.395	0.03631	*	−1.674	Liao et al. (2020)
		6 weeks	−0.676	0.00112	**	−1.057	
CCL23	P55773	4 weeks	−0.261	0.08495		−1.056	Simats et al. (2018)
CCL3	P10147	2 weeks	−1.275	0.04930	*	−1.455	Pelisch et al. (2020)
CXCL10	P02778	2 weeks	−0.473	0.02959	*	−1.003	Landreneau et al. (2018)
		6 weeks	−0.380	0.07343		−1.340	
IL18	Q14116	2 weeks	−0.221	0.01250	*	−1.213	Hao et al. (2019)
		4 weeks	−0.180	0.03597	*	−1.609	
IL6	P05231	2 weeks	−0.570	0.02281	*	−1.314	Mosarrezaii et al. (2020), Zhu et al. (2022)
		6 weeks	−0.555	0.02615	*	−1.253	
MCP-1	P13500	2 weeks	−0.500	0.00435	**	−1.644	Ho et al. (2012)
		4 weeks	−0.338	0.04056	*	−1.318	
		6 weeks	−0.375	0.02511	*	−1.116	
PD-L1	Q9NZQ7	4 weeks	−0.411	0.08409		−1.017	Bodhankar et al. (2015)
TNF⍺	P01375	2 weeks	−0.543	0.06626		−1.264	Shohami et al. (1999), Longhi et al. (2013)
		4 weeks	−0.553	0.06171		−1.403	
		6 weeks	−0.531	0.07164		−0.959	
TNFβ	P01374	2 weeks	−0.370	0.01309	*	−1.457	Stahel et al. (2000)
TNFRSF9	Q07011	2 weeks	−0.359	0.02215	*	−1.194	He et al. (2018)
TRAIL	P50591	4 weeks	−0.354	0.05339		−1.318	Hoffmann et al. (2009)
Anti-inflammatory							
CX3CL1	P78423	2 weeks	−0.503	0.00232	**	−1.464	Lauro et al. (2019), Pawelec et al. (2020)
		4 weeks	−0.331	0.03171	*	−1.411	
IL10Rβ	Q08334	2 weeks	−0.210	0.03186	*	−1.564	Garcia et al. (2017)
		4 weeks	−0.317	0.00249	**	−1.269	
LIF-R	P42702	2 weeks	−0.213	0.06464		−1.073	Davis and Pennypacker (2018)
NT3	P20783	2 weeks	−0.477	0.00038	***	−1.530	Lin et al. (2021)
		4 weeks	−0.251	0.03390	*	−1.279	
		6 weeks	−0.214	0.06566		−1.445	
TGF⍺	P01135	2 weeks	−0.287	0.06589		−1.415	Dai et al. (2020)
uPA	P00749	2 weeks	−0.352	0.03341	*	−1.223	Merino et al. (2017)
VEGF⍺	P15692	2 weeks	−0.231	0.07128		−1.035	Li et al. (2016), Geiseler and Morland (2018)
		4 weeks	−0.244	0.05780		−1.220	

Group differences in both pro- and anti-inflammatory biomarkers normalized to baseline. The protein biomarker, the UniProt.org ID for each protein, the timepoint of the measurement, the group differences (MSC-EV treated mean - vehicle control mean), adjusted *P*-value, significance, and Cohen’s d standard deviational unit effect size are listed. A two-way ANOVA was used to assess treatment by timepoint analysis, and a Tukey’s post-hoc analysis was used to identify significant differences. The complete data set can be found in Supplementary Table S5A/B. ***p < 0.001, **p < 0.01, *p < 0.05.

Microglial cell densities were compared using Student’s t-tests with corrections for multiple comparisons in GraphPad Prism 10 to analyze between-group differences. Proportions of microglial phenotypes in 6 and 16 week recovery cohorts (see Section 2.1) were compared using 2-way ANOVA (treatment x cohort). Measures of microglial phenotype density were correlated with plasma biomarkers and days to return to pre-operative grasp, using linear regression based on Pearson’s correlation.

To initially assess the relationship between recovery metrics and inflammatory biomarkers in blood, CSF and brain tissue, linear regression analysis based on Pearson’s correlation (R) was used (GraphPad Prism 10). Due to the small sample size and the large number of biomarkers analyzed, each outcome measure was treated as independent. Further, multivariate analyses of the Olink plasma biomarkers full dataset (64 markers) were done in MATLAB (version 2024a, Natick, MA, USA). We performed principal component analyses for dimensional reduction and assessed the relative contribution of each biomarker to the variance in the dataset. To assess the relative (dis)similarities across monkeys and timepoints, we performed non-parametric hierarchical clustering by monkey (n = 4 per group) and timepoint (*n* = 5 timepoints per monkey) based on the mean concentration (per timepoint/monkey) of 64 plasma proteins measured with Olink® PEA as described (Medalla et al., 2023). We normalized data to z scores, then calculated a distance matrix based on pairwise calculation of Euclidean distances between each datapoint. Hierarchical clustering analysis (HCA) based on these pairwise distances was performed, based on our previous work (Medalla et al., 2023).

#### 2.12.2 Biological pathway analysis of plasma protein biomarkers

To assess the biological pathways that are potentially associated with MSC-EV treatment, we performed a functional annotation analysis of biological pathways associated with the differentially expressed proteins (DEP) between treatment groups, using the NIH Database for Annotation, Visualization, and Integrated Discovery (DAVID v6.8) (Huang et al., 2009a,b; Li et al., 2022). The DEPS from the 2-week time point were uploaded using Uniprot ID into the DAVID tool. A list of terms from the gene ontology (GO) (The Gene Ontology Consortium) molecular functions, cellular components, and biological processes databases (Ashburner et al., 2000; Aleksander et al., 2023) and the Kyoto Encyclopedia of Genes and Genomes (KEGG) (Kanehisa Laboratories) (Kanehisa and Goto, 2000) pathway database related to the DEPs was generated. The significantly enriched terms were selected based on fold-enrichment value of >1, a *p* < 0.05 (with Benjamini correction for multiple comparison) and a false discovery rate (FDR) value < 0.05.

## 3 Results

### 3.1 Recovery of fine motor function

As summarized in Figure 1A, aged female monkeys were assessed for the effects of MSC-EV treatment on the recovery of fine motor hand function, 4–6 weeks after induction of cortical injury to the hand representation of M1 (Figures 1A,B). The aged female monkeys in the current study exhibited patterns of functional recovery after injury and treatment, consistent with our previous study (Moore et al., 2019). Specifically, between-group comparisons revealed that MSC-EV treated monkeys took fewer mean number of days to recover pre-operative grasp function or reach a plateau in recovery compared to vehicle monkeys (Student’s *t*-test, *p* = 0.031, Figure 1C). In contrast, vehicle monkeys exhibited persistent compensatory grasp patterns and reached a recovery plateau, not returning to pre-operative grasp function (Figure 1C). Similarly, the mean grasp pattern in the first week of post-operative testing (3 weeks post injury) in the current cohort revealed a similar trend to our previous cohort, with higher grasp rating in MSC-EVs compared to vehicle monkeys but did not reach statistical significance (Mann–Whitney Test, *p* = 0.086, Figure 1D). Notably, 3 of the 4 MSC-EV treated animals in the current cohort demonstrated a complete return to preoperative grasp function (a score of 8 on the GRAS) in the 1st week of testing, while none of the vehicle control monkeys showed this level of recovery (Figure 1D). Across the whole 6 week recovery period, we did not find a significant difference in the mean grasp rating (Mann–Whitney Test, *p* = 0.20, Figure 1E); However, pooling the data from the current and previous cohort (Moore et al., 2019) revealed consistent significant between-group differences across all measures of functional recovery (Moore et al., 2019). Specifically, we found that when considering this larger cohort of monkeys, significant between-group differences (*p* < 0.05) were found in the mean number of days to recover, as well as post-operative grasp rating in both the first week of post-operative testing, and the mean across the first 4 weeks of the recovery period (Supplementary Figure 2).

### 3.2 Effect of injury on the temporal changes in inflammatory plasma biomarkers across recovery

**Figure 2:**
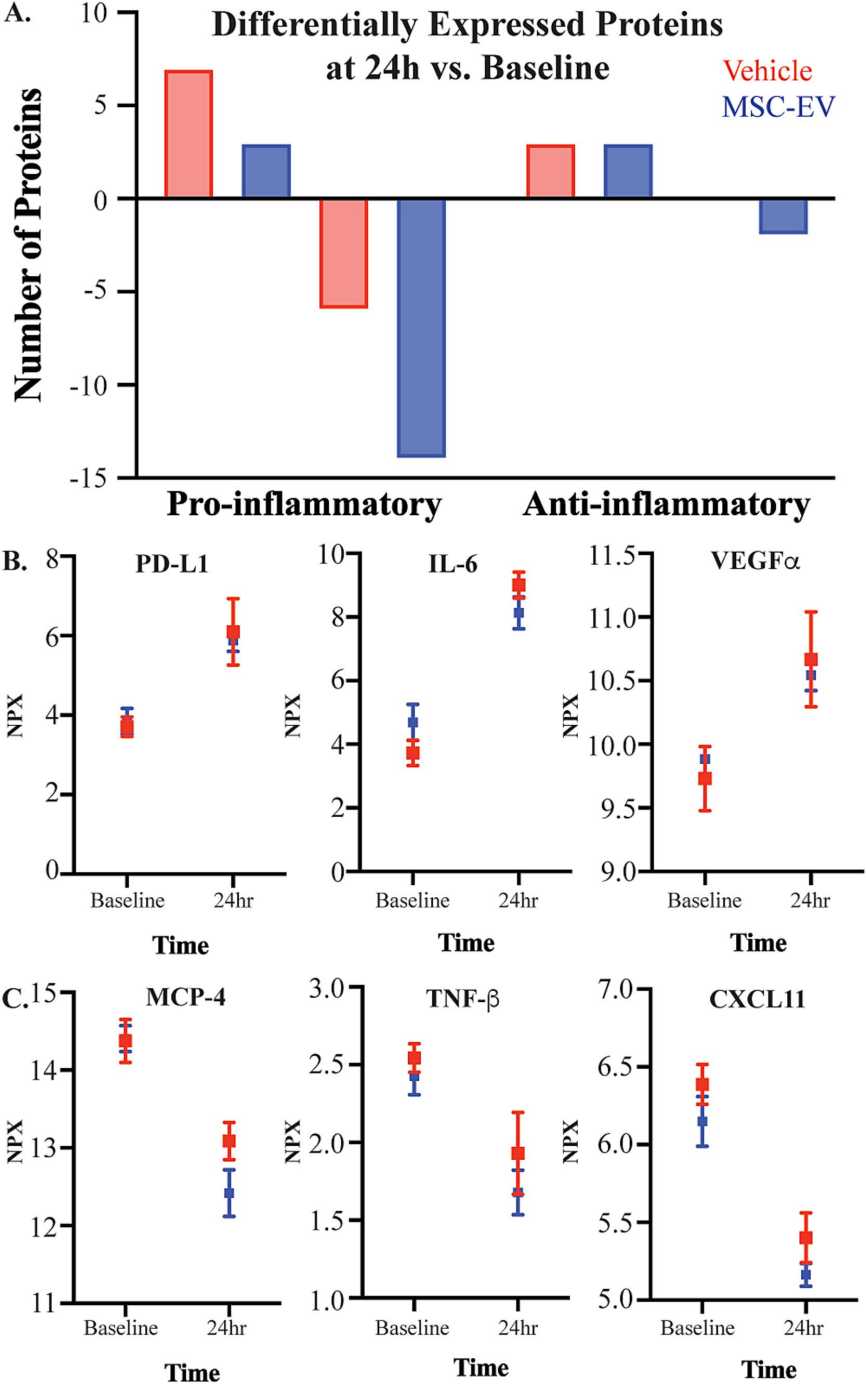
The effect of injury on plasma inflammatory biomarkers. Concentration of inflammatory mediators in plasma were measured with the Olink^®^ PEA, with all values presented as raw NPX log2 values. **(A)** The number of canonically pro- and anti-inflammatory proteins that were differentially up-or down-regulated 24 h post-injury relative to baseline, in plasma of MSC-EV (*n* = 4) and vehicle (*n* = 4) monkeys. **(B)** Representative graphs of significantly upregulated proteins PDL-1, VEGFα, and IL-6, and **(C)** significantly downregulated proteins MCP-4, TNF-*β*, and CXCL11 at 24 h post-injury relative to baseline.

To understand the biological effects of lesion and MSC-EV treatment across recovery, we collected blood samples at the baseline, 24-h, 2-week, 4-week, and 6-week time points across recovery. Using the Olink^®^ PEA, levels of inflammatory proteins, cytokines, chemokines, neurotrophins, and growth factors in plasma were quantified. First, we assessed the effect of lesion in both the MSC-EV and vehicle treated groups, comparing raw NPX expression values of each biomarker between groups and timepoints (two-way ANOVA, treatment x timepoint, with Fisher’s LSD post-hoc; Figure 2; Supplementary Table S3). Largely, we found that the groups were similar in inflammatory plasma profiles at baseline and at 24 h post-injury, prior to treatment, with only a small subset of inflammatory biomarkers demonstrating between group differences at the baseline (CCL20, uPA, CXCL10, Supplementary Table S3) and at 24-h (CCL20, CASP-8, IL10-Rβ, CD40, Supplementary Table S3).

We then assessed the changes of plasma biomarkers across recovery, comparing raw expression values between-timepoints, within each group (Figure 2; Table 1; Supplementary Table S3). Significant differences in inflammatory plasma proteins were found between the 24-h post-injury timepoint and other timepoints, in both vehicle and MSC-EV treated animals (Supplementary Table S3). Specifically, we found a high number of differentially expressed proteins (DEPs) at 24 h post-injury relative to baseline, indicating a significant effect of injury on the inflammatory profile of plasma, which was consistent between the treatment groups (Figure 2A; Supplementary Table S3). Overall, the majority of DEPs at 24 h post-injury vs. baseline were canonical pro-inflammatory proteins (Figure 2A; Table 1). At 24 h post-injury, but prior to MSC-EV or vehicle treatment, we found a significant upregulation of a subset proteins compared to baseline in both groups, including IL-6, VEGF⍺, PD-L1 (Figure 2B; Supplementary Table S3). Also at 24 h, we found that there was a significant downregulation of CXCL11, MCP-4 and TNFβ (Figure 2C; Table 1; Supplementary Table S3), at 24 h as compared to baseline. These DEPs all have important roles in the post-injury inflammatory response, VEGF⍺ is a growth factor that has context-dependent beneficial effects of angiogenesis and detrimental effects on the blood brain barrier (Li et al., 2016; Geiseler and Morland, 2018), while IL-6 is a cytokine with a dual role as a pro-inflammatory molecule related to more severe stroke (Mosarrezaii et al., 2020; Zhu et al., 2022) and possesses neurotrophic properties (Zhu et al., 2022). MCP-4 is a chemoattractant protein that targets multiple inflammatory cells (Lamkhioued et al., 2000).

In addition to DEPs between 24-h post-injury and baseline, we found a subset of proteins that were differentially expressed at 2 weeks, 4 weeks, and 6 weeks post-injury compared to 24-h timepoint in both groups (Supplementary Table S3). Interestingly, we observed a divergence in the temporal patterns of recovery-associated plasma profiles of the two groups at 2 weeks, when significant DEPs between treatment groups were found (Table 1). Further, in the vehicle group, but not the MSC-EV group, we found significant differential expression of inflammatory markers at 2 weeks relative to baseline (Table 1; Supplementary Table S3). These differences from baseline at 2 weeks were not observed to the same degree in the MSC-EV group. These data indicate a treatment effect on the time course of plasma inflammatory profiles, consistent with a sustained inflammatory environment in the vehicle control animals, and an earlier shift towards a homeostatic environment in the MSC-EV animals.

### 3.3 MSC-EV treatment is associated with a lower expression of inflammatory biomarkers in plasma

**Figure 3:**
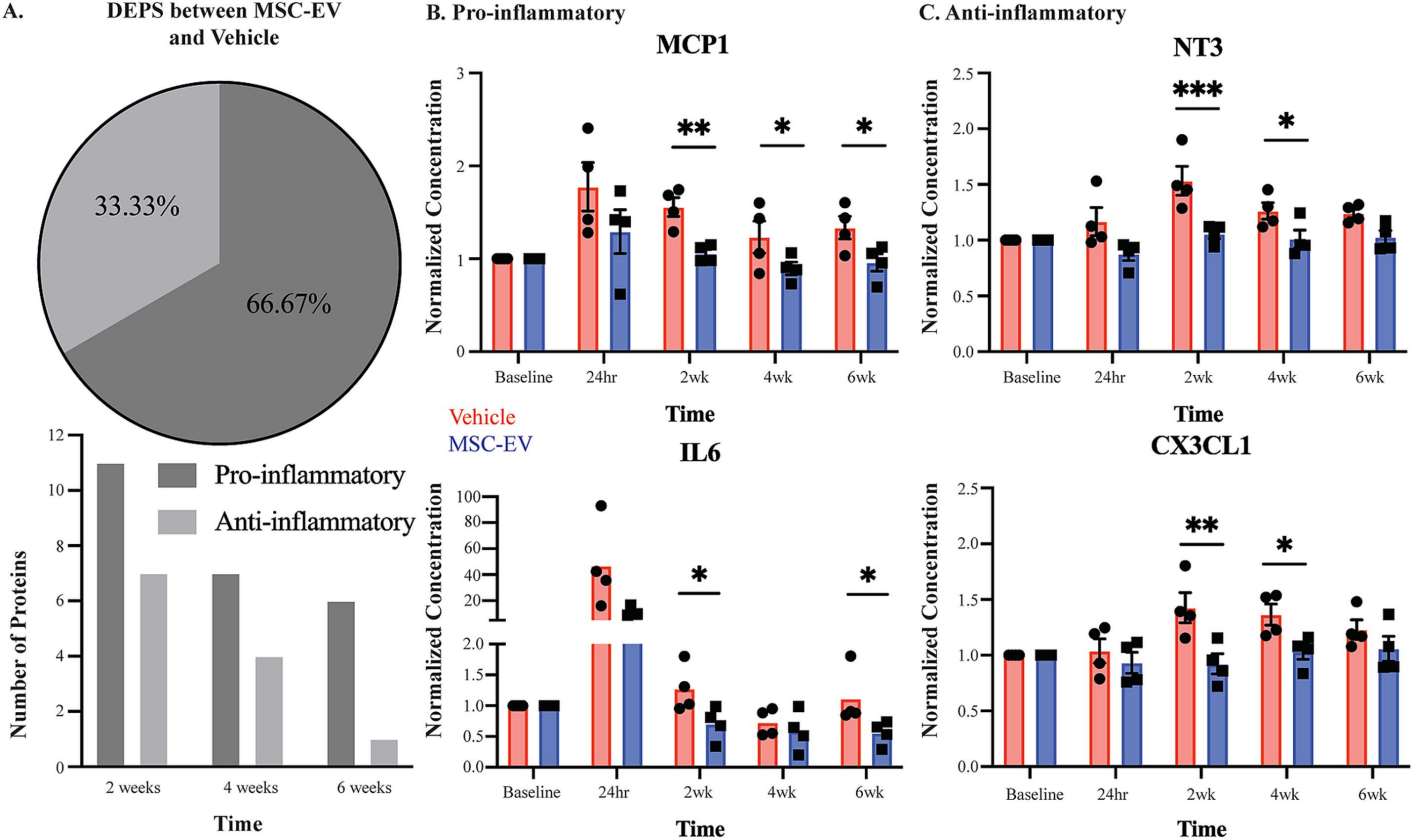
Measures of plasma inflammatory biomarkers across recovery after cortical injury. Concentration of inflammatory mediators in plasma across recovery were measured with the Olink_®_ PEA, with all values normalized to the baseline measurements. **(A)** Number of DEPs between MSC-EV vs. Vehicle monkeys at each timepoint, and the proportion of these DEPs that are canonically pro- or anti-inflammatory. **(B,C)** Normalized concentration of a subset of pro-inflammatory mediators MCP1, and IL6 and anti-inflammatory mediators CX3CL1 and NT3, which were differentially expressed between groups. MSC-EV (*n* = 4) and vehicle (*n* = 4); ***p < 0.001, ** *p* < 0.01, *p < 0.05.

To isolate the effect of MSC-EV treatment at each post-injury timepoint on inflammatory proteins, we normalized values at each timepoint to baseline values to account for inter-subject variability. Two-way repeated measures ANOVA with treatment x timepoint as independent variables was employed on post-treatment timepoints (2 weeks, 4 weeks, 6 weeks) (Supplementary Table S5A). All *p*-values reported were adjusted for multiple comparisons, and the effect sizes were reported as Cohen’s d in standard deviational units (Table 2; Supplementary Table S5B). There were significant effects of treatment for multiple inflammatory plasma proteins at all post-treatment timepoints, but mainly at 2 weeks post-injury. Overall, the data revealed a significant downregulation of a subset of both pro- and anti-inflammatory proteins with treatment (Table 2; Figure 3). However, the majority (67%) of the proteins downregulated across all timepoints were canonically pro-inflammatory (Figure 3A). The remainder of the downregulated proteins, which were canonically anti-inflammatory, were downregulated mostly at 2 weeks post injury (Figure 3A). At 2 weeks post-injury, the numbers of downregulated pro- and anti-inflammatory proteins were the highest relative to other timepoints. At 4 weeks and 6 weeks post-injury, a relatively high number of downregulated pro-inflammatory proteins persisted, while the number of downregulated anti-inflammatory proteins declined (Figure 3A).

Between-group post-hoc comparisons at each timepoint (Tukey’s HSD) revealed that plasma from MSC-EV treated monkeys exhibited significantly lower levels of a subset of pro-inflammatory markers throughout the recovery period (Table 2 for downregulated proteins, Cohen’s d effect size, *p*-value; Supplementary Table S5B for full dataset). The following pro-inflammatory markers were downregulated significantly (*p* < 0.05) or approached significance (*p* < 0.10) (Table 2) in MSC-EV vs. Vehicle group only at 2 weeks post injury: CCL3, a chemoattractant protein that contributes to secondary damage in spinal cord injury (Pelisch et al., 2020). TNFβ (also known as lymphotoxin alpha) and TNFRSF9 (also known as CD137), which activate downstream inflammatory pathways, increasing inflammation (Stahel et al., 2000; He et al., 2018) and CCL19, a protein involved in arteriole growth following ischemia (Nossent et al., 2017) that is correlated with worse outcome following stroke (Che et al., 2023).

A substantial subset of pro-inflammatory markers relevant to brain injury was downregulated in MSC-EV vs. vehicle monkeys, at multiple timepoints across the 2–6 week post-injury recovery time window. In particular, we found CCL11, MCP1, and TNF⍺, which are robust pro-inflammatory mediators that impair recovery following cortical injury while driving secondary damage (Lieschke et al., 2019; Ho et al., 2012; Shohami et al., 1999; Longhi et al., 2013), downregulated in the MSC-EV vs. vehicle monkeys at all three timepoints. Similarly, IL-18 was downregulated in MSC-EV vs. vehicle monkeys at 2 weeks and 4 weeks, and CXCL10 at 2 weeks and 6 weeks post-injury. Both proteins are pro-inflammatory cytokines that can induce inflammatory cell activity and are higher in humans with worse outcome following stroke (Hao et al., 2019; Landreneau et al., 2018). In addition, IL-6 and CCL20, which are pro-inflammatory mediators linked to microglial activation and microglial-mediated neurodegeneration in mouse models of cortical injury (Liao et al., 2020; Mosarrezaii et al., 2020; Zhu et al., 2022), were downregulated in MSC-EV vs. vehicle at 2 weeks and 6 weeks post-injury. A number of pro-inflammatory proteins were downregulated in MSC-EV vs. vehicle monkeys only at the 4-week timepoint. These include PD-L1 and TRAIL, two pro-inflammatory proteins whose downregulation has beneficial effects in models of stroke (Bodhankar et al., 2015; Hoffmann et al., 2009) and CCL23, a chemokine that induces further expression of inflammatory proteins in a feed-forward mechanism (Simats et al., 2018). Representative plots of pro-inflammatory proteins MCP1 and IL-6 across recovery are presented (Figure 3B).

Compared to the pro-inflammatory markers, there were fewer anti-inflammatory markers downregulated with MSC-EV treatment. Further, most of these downregulated anti-inflammatory markers in the MSC-EV treated group were significantly different only at 2 weeks post-injury, early in the recovery timeline and before the second MSC-EV treatment. There was a smaller subset of anti-inflammatory proteins that were also downregulated in MSC-EVs vs. vehicle group at 4 weeks post-injury (Table 2). Specifically, at 2 weeks, both TGF⍺ and LIF-R, two proteins involved in post-injury neuroprotection (Dai et al., 2020; Davis and Pennypacker, 2018), had lower levels in MSC-EVs vs. vehicle monkeys, along with urokinase-type plasminogen activator (uPA), a protein involved in axonal repair mechanisms (Merino et al., 2017). In addition, the following markers were downregulated in MSC-EV vs. vehicle monkeys both at 2 weeks and 4 weeks post injury: CX3CL1 and IL10Rβ, anti-inflammatory proteins that are involved in neuroprotection and repair following cortical injury (Lauro et al., 2019; Pawelec et al., 2020; Garcia et al., 2017) and VEGF⍺ (Li et al., 2016; Geiseler and Morland, 2018). Only one anti-inflammatory protein was downregulated at all timepoints post-injury: NT3, a growth factor associated with neuroprotection (Lin et al., 2021). Representative plots of anti-inflammatory proteins CX3CL1 and NT3 across recovery are presented (Figure 3C).

Overall, these results suggest that MSC-EV treatment is associated with a general suppression of the immune response, mainly 2-weeks following cortical injury, by modulating the levels of pro- and anti-inflammatory plasma biomarkers. Further, while MSC-EV-associated downregulation of anti-inflammatory proteins was predominant at 2 weeks post-injury, the downregulation of pro-inflammatory proteins persisted across the 2 to 6-week post-injury recovery period. These data demonstrate that as recovery progresses, the effect of MSC-EVs shifts from an acute suppression of overall inflammatory markers to a more selective suppression of pro-inflammatory plasma markers during more chronic recovery timepoints.

### 3.4 Multivariate analyses and functional annotation analyses of inflammatory profiles associated with treatment and injury

**Figure 4:**
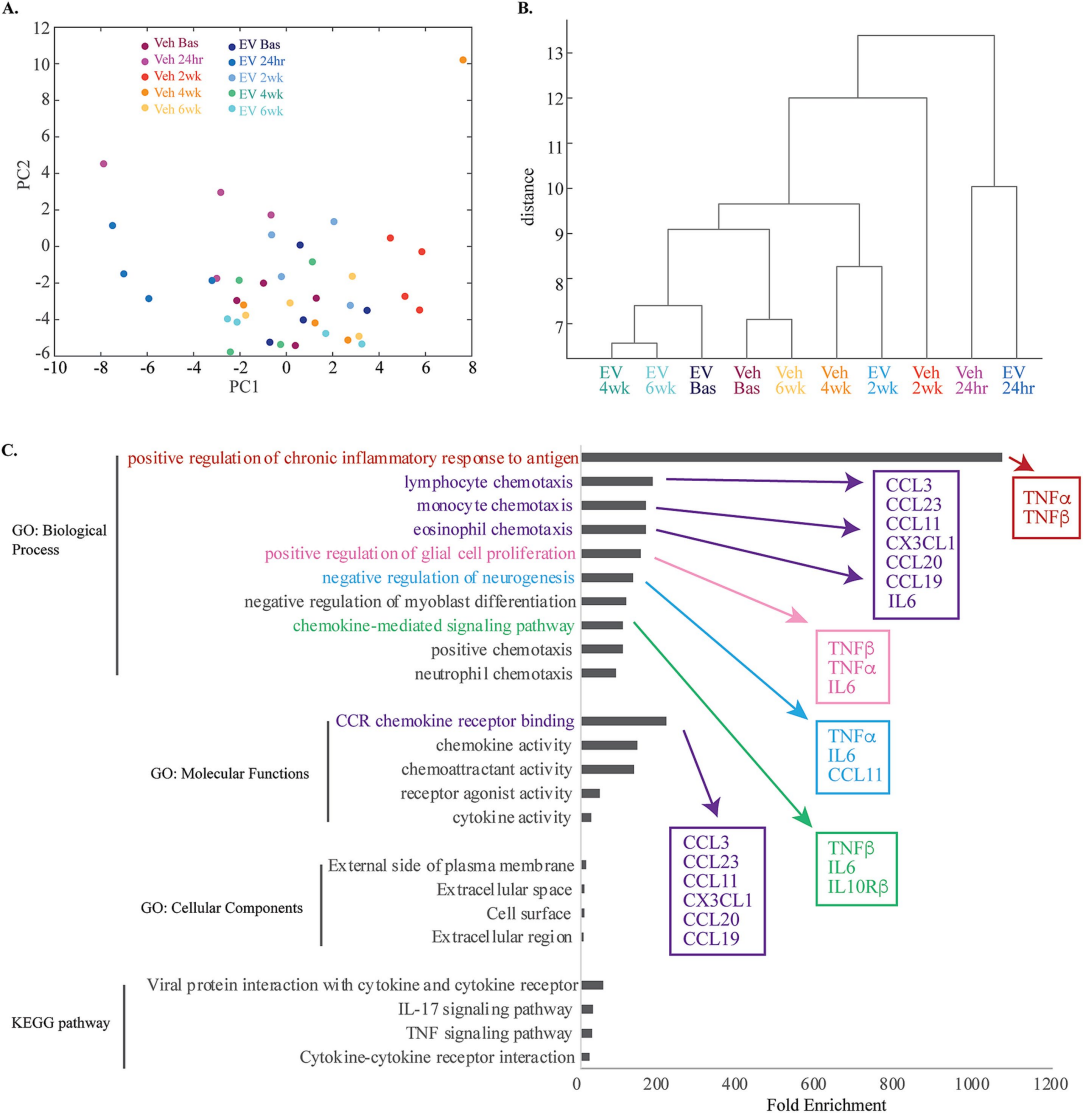
Multivariate and functional annotation analyses of inflammatory biomarkers in plasma associated with treatment and injury. **(A)** Principal component analyses of 64 total plasma biomarkers, revealed 18 PCs explaining 95% of the total variance. The scatter plot of the first two PCs, which explained 20 and 15% of the variance, respectively are shown (see Supplementary Table S6). Note the clustering of 24-h timepoint from both groups, and 2-week timepoint from the vehicle group. **(B)** Hierarchical clustering analyses dendrogram using complete (farthest distance) linkage calculated from a distance matrix of Euclidean distances based on the total 64 Olink inflammatory biomarker expression profiles. **(C)** A horizontal bar plot of fold enrichment of significantly enriched GO and KEGG pathway terms (adjusted *p* values < 0.05, FDR < 0.05) resulting from DAVID functional annotation analysis on the subset of significantly downregulated proteins in MSC-EV versus vehicle group (see Supplementary Table S7).

To further investigate the multivariate effects of treatment and timepoint on inflammatory protein expression profiles, we performed principal component analyses and dimensional reduction of our full 64-marker Olink dataset (Figure 4, Supplementary Table S6). The analyses revealed that 18 Principal Components (PC) explained 95% of the variance in the dataset. The first two PCs explained 20% and 15% of the variance, respectively. The variables that contributed most to the variance of the top 9 PCs include many of the differentially expressed markers we assessed (Figures 4A,B, Supplementary Table S6). Plotting principal components 1 and 2 shows some segregation of data points from the 24-h timepoint from both groups, and 2-week timepoint from the vehicle group, clustering away from the rest of the datapoints (Figure 4A). This was confirmed with non-parametric HCA, assessing the relative (dis)similarities across cases and timepoints based on Olink inflammatory biomarker expression profiles (Figure 4B). We found that 24-h timepoint initially clustered separately and was the most different (largest distance) from the rest of the subgroups, demonstrating the early effects of the lesion causing the greatest disruption to the inflammatory environment. The next node of clustering segregated 2-week Vehicle from the rest of the data. Interestingly, the next clustering node subclustered Vehicle 4-week and MSC-EV 2-week timepoints, distinct from the other groups. The remaining cluster consist of baseline and 6-week data from both groups and 4-week data from the MSC-EV group, which had high relative similarity to each other (small distances). These findings are indicative of the MSC-EV treatment accelerating the shifts in peripheral inflammatory plasma profiles towards a more homeostatic, baseline state, while the profile of vehicle control monkeys remain in a chronic state of inflammation (more dissimilar from baseline values), for a more prolonged period following injury.

To understand the functional relevance of the inflammatory biomarkers affected by treatment, we performed functional annotation (Gene Ontology and KEGG pathway) analyses on the subset of DEPs (all of which were downregulated at 2-weeks post injury) in MSC-EV versus vehicle group (DAVID v6.8) (Huang et al., 2009a,b) (Proteins presented in Table 2 and pathway analyses in Figure 4C and Supplementary Table S7). Analysis of these proteins yielded a list of functional terms associated with an upregulation of immune response (Figure 4C, Supplementary Table S7). Some of the highest significantly enriched pathways include: positive regulation of inflammatory response to antigen, lymphocyte chemotaxis, monocyte chemotaxis, eosinophil chemotaxis, positive regulation of glial cell proliferation, negative regulation of neurogenesis, and chemokine mediated signaling pathway (Figure 4C, Supplementary Table S7). Thus, these analyses show that MSC-EV treatment after cortical injury is associated with a downregulated peripheral immune response and inflammatory cascades. Interestingly, some functions related to negative modulation of plasticity and repair (negative regulation of neurogenesis) were also associated with the MSC-EV mediated protein downregulation (Figure 4C) and thus decreases in these proteins could be beneficial for neurite growth and recovery. In summary, these functional annotation analyses suggest that the downregulation of inflammatory proteins with MSC-EV treatment following cortical injury can lead to dampening of the peripheral immune response as well as direct suppression of the negative modulation (hence promotion) of plasticity and repair.

### 3.5 Decreased peripheral inflammatory biomarkers correlated with functional recovery

**Figure 5:**
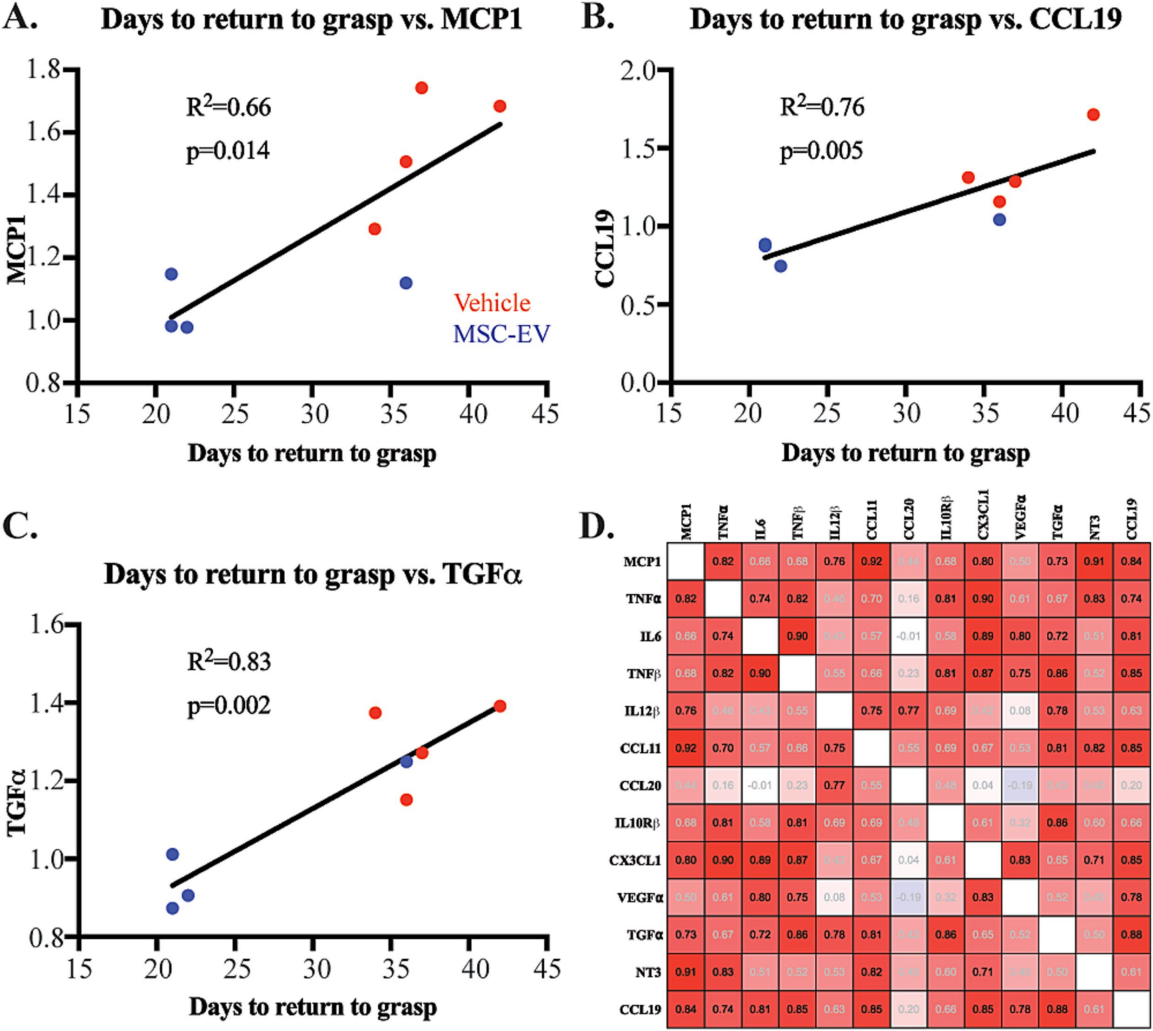
Correlation of plasma inflammatory biomarkers with functional metrics of recovery after cortical injury. **(A-C)** Scatter plot and linear regression showing significant correlations of days to return to pre-operative grasp with normalized plasma concentrations of inflammatory biomarkers MCP1, CCL19, and TGFα at 2-weeks post injury: Vehicle control monkeys (red), MSC-EV monkeys (blue). **(D)** Correlation matrix of pro- and anti-inflammatory proteins at 2 weeks post-injury, showing predominantly positive correlations (Red) between most inflammatory proteins. Pearson’s R value is listed is within each square: black text p < 0.05, gray text *p* > 0.05.

We then assessed how the inflammatory changes observed in plasma biomarkers (specifically at the 2-week timepoint) correlate with measures of recovery using linear regression based on Pearson’s correlation. We found that lower plasma levels of inflammatory biomarkers at 2 weeks post-injury were significantly correlated (*p* < 0.05) with enhanced recovery of grasp function post-injury (fewer days to return to pre-operative grasp patterns; Figure 5). Figure 5 shows individual scatter plots with linear regression of a subset of significantly correlated biomarkers and functional measures. The number of days to return to grasp was positively correlated with plasma levels of the following biomarkers at 14-days post-injury: pro-inflammatory MCP1 (R^2^ = 0.662, *p* = 0.014, Figure 5A), CCL19 (R^2^ = 0.764, *p* = 0.005, Figure 5B), CCL11 (R^2^ = 0.793, *p* = 0.003, data not shown), and IL12β (R^2^ = 0.684, *p* = 0.0113, data not shown); anti-inflammatory TGF⍺ (R^2^ = 0.831, *p* = 0.002, Figure 5C).

Further, we assessed the correlation between inflammatory markers at the 2-week timepoint, using a Pearson’s correlation matrix. We found significant positive correlations (*p* < 0.05, Supplementary Table S8) of pro-inflammatory biomarkers with both other pro- and anti-inflammatory markers (Figure 5D). For instance, the major proinflammatory signal, TNF⍺ was significantly positively correlated with MCP1, IL6 and TNFβ. MCP1 is additionally correlated with IL12β, CCL11, CCL19 (Figure 5D, *P*-values presented in Supplementary Table S8). Similarly, anti-inflammatory CX3CL1 showed significant correlation with anti-inflammatory factor NT3 (p < 0.05). Our data also showed a strong positive correlation between several pro- and anti-inflammatory markers. For example, canonically anti-inflammatory marker CX3CL1 was significantly positively correlated with pro-inflammatory markers (MCP1, TNF⍺, IL6, TNFβ; Figure 5D). These correlations suggest a complex balance between plasma inflammatory biomarkers after injury. In summary, these data further support how MSC-EV treatment may lead to a general decrease in the expression of plasma inflammatory biomarkers, which can alter the time course of complex pro- and anti-inflammatory signaling to support functional recovery after cortical injury.

### 3.6 MSC-EV treatment does not lead to changes in the inflammatory profile in CSF

**Figure 6:**
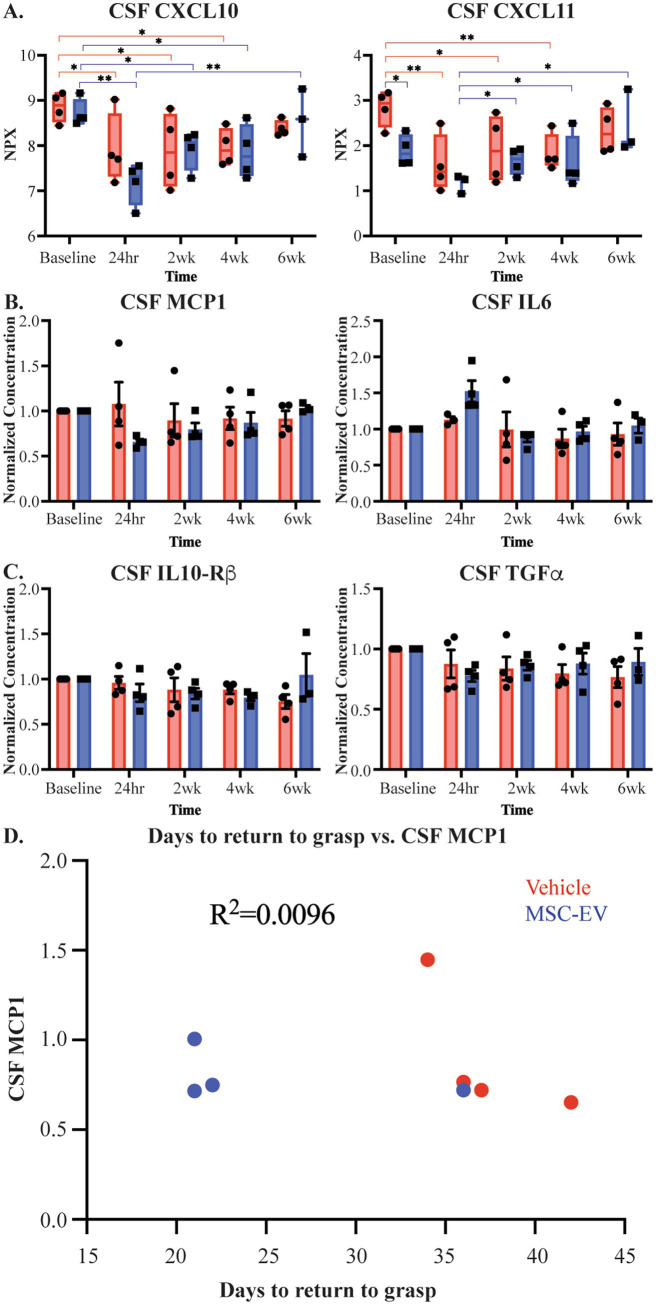
Measures of CSF inflammatory biomarkers across recovery after cortical injury. **(A)** Measurements of CSF inflammatory proteins raw NPX values across recovery for CXCL11 and CXCL10. Brackets indicate pairs with significant between-group (black) and between-timepoint (MSC-EV: blue, vehicle: red) differences. **(B,C)** Normalized measurements of pro-inflammatory biomarkers IL6 and MCP1 in CSF and anti-inflammatory biomarkers TGFα and IL-10Rβ in CSF. **(D)** Representative scatter plot showing lack of correlations of days to return to pre-operative grasp and inflammatory biomarker MCP1 in CSF at 2 weeks post-injury (MSC-EV: blue, vehicle: red). MSC-EV (*n* = 4) and Vehicle (*n* = 4). **p < 0.01, *p < 0.05.

Our previous studies have demonstrated the efficacy of MSC-EVs in ameliorating the consequences of cortical injury in brain tissue, at 16 weeks post-injury (Moore et al., 2019; Go et al., 2020; Medalla et al., 2020; Go et al., 2021; Calderazzo et al., 2022; Zhou et al., 2023), a later recovery timepoint than that of the current study. Since the treatment was given via IV infusion, whether the effect of MSC-EV treatment on brain tissue is direct or via peripheral mechanisms that are reflected in changes in blood and CSF remains unclear. Thus, we analyzed inflammatory profiles of CSF samples collected in parallel to plasma samples. Analysis of CSF samples from monkeys in this study using the Olink^®^ PEA yielded results for 39 proteins, 23 of which were of interest in cortical injury (Supplementary Table S9). In contrast to plasma, two-way ANOVA comparisons of raw expression values, by treatment x timepoint, revealed largely no significant differences for the majority of markers, except for in a few proteins (Timepoint main effect, CXCL10 *p* = 0.001, CXCL11 *p* = 0.004, and CCL11 *p* = 0.024, Group main effect, CXCL11 *p* = 0.049, CD40 *p* = 0.013 representative examples Figure 6A; Supplementary Table S10). Similarly, comparisons of normalized values (normalized to baseline) of these CSF biomarkers at post-treatment timepoints (2-, 4- and 6-weeks post-injury; two-way repeated measures ANOVA, treatment x timepoint) revealed no significant effects of treatment or timepoint (Figures 6B,C; Supplementary Table S12). Unlike the pattern seen with plasma biomarkers, there were no correlations between levels of inflammatory proteins in CSF and functional recovery (representative example, Figure 6D).

### 3.7 MSC-EV treatment altered microglial morphology and MHCII expression

**Figure 7:**
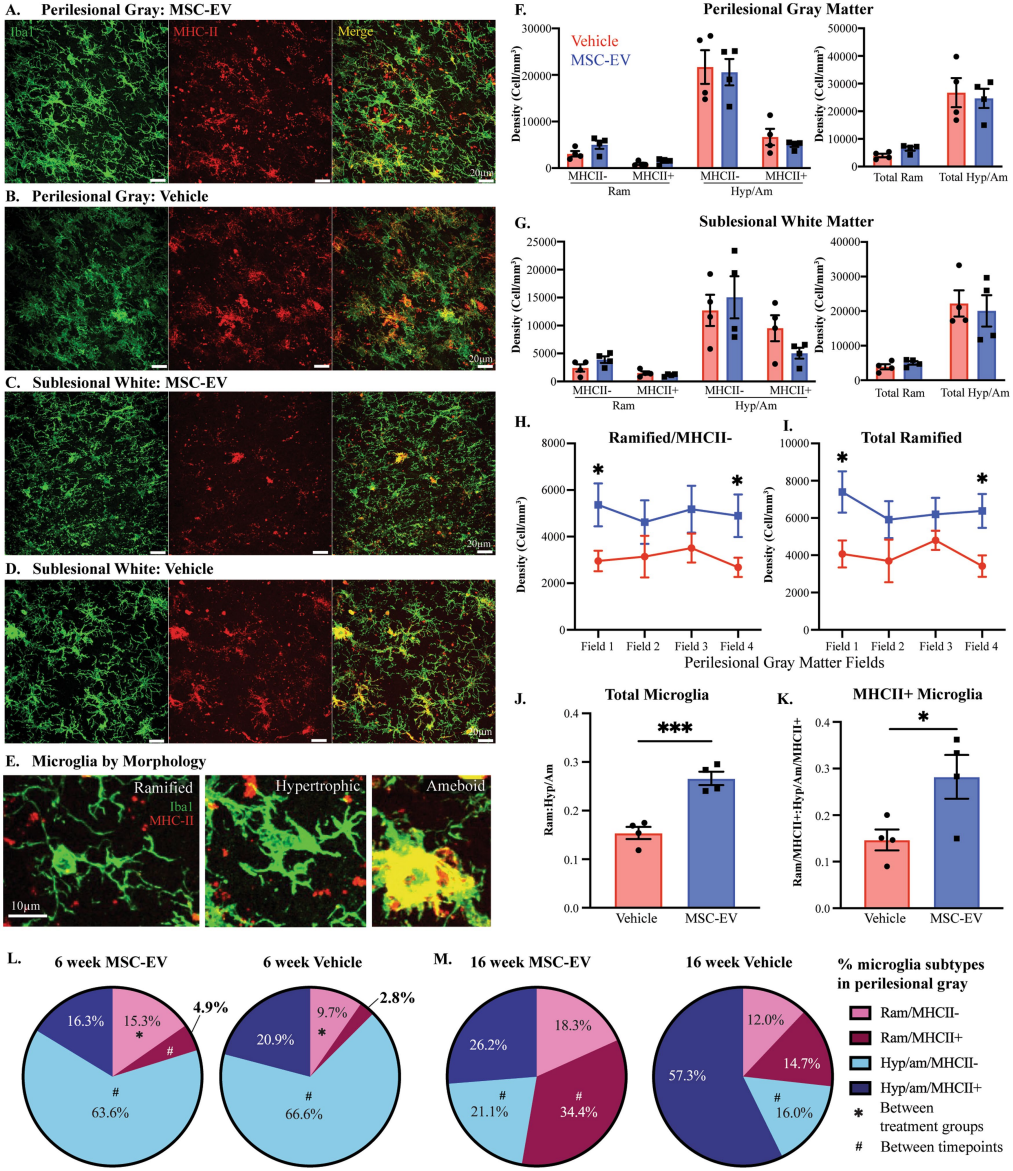
Microglial inflammation in the perilesional cortex across 6- and 16-week post-injury. **(A-D)** Representative images (maximum z projection, 30 µm) of immunofluorescent staining of Iba1/P2RY12 (Green) and MHC-II (Red) in the perilesional gray matter and sublesional white matter. **(E)** Images showing examples of microglia subtypes based on morphology and MHCII expression: Ramified/MHCII- (left panel), Hypertrophic/MHCII- (middle panel) and Amoeboid/strongly MHCII+ (right panel) microglia. **(F,G)** Density of microglial by morphology (Ram: ramified, Hyp/Am: hypertrophic/ameboid) and MHCII expression, and by morphology only, quantified via 3D stereological counting methods in perilesional gray matter **(F)** and sublesional white matter **(G)**. **(H)** The density of ramified/MHCII- microglia and **(I)** total ramified microglia by field (distance from lesion) in perilesional gray matter. **(J)** Ratio of ramified:hypertrophic/amoeboid for total microglia and **(K)** the subset expressing MHCII+ in perilesional gray. **(L, M)** Pie charts showing the percentage of each microglial phenotype in the perilesional gray matter at 6-weeks post-injury (**L**, data from current cohort; MSC-EV *n* = 4 and vehicle *n* = 4) compared to 16 weeks post-injury (**M**, data adapted from Go et al., 2020; MSC-EV *n* = 4 and vehicle *n* = 5). Between-group: ***p < 0.001, *p < 0.05. Between-timepoint: #*p* < 0.05.

Our previous analyses of brain tissue harvested at 16 weeks post-injury indicate effects of MSC-EVs on the phenotypes of microglia, the major immune cells of the brain (Go et al., 2020; Zhou et al., 2023). Thus, we investigated whether these microglia differences are evident at an earlier timepoint, 6-weeks post injury, and if any changes are related to peripheral inflammatory changes and functional recovery. We analyzed perilesional cortex from the brain tissue harvested 6 weeks post-injury and quantitatively assessed injury and treatment-related changes in microglial morphology (labeled with Iba1) and expression of MHCII, a marker of immune-activated, antigen presenting microglia when colocalized with Iba1 (Schetters et al., 2017). Images from perilesional motor cortex and sublesional white matter were acquired radially beginning 200 μm from the lesion and each subsequent field 400 μm away from the previous field, towards the white matter (Imaging methodology presented in Figure 1B). Representative images from both perilesional gray and sublesional white matter are presented for both MSC-EV treated and vehicle control monkeys (Figures 7A–D).

The density and proportion of distinct microglia phenotypes based on morphology and expression of MHCII (Schetters et al., 2017) were quantified using 3D counting methods, as described previously (Tsolias and Medalla, 2021; Tsolias et al., 2024). Microglia were classified based on MHCII expression as well as their “ramified” versus “hypertrophic/ameboid” morphology, as defined in Karperien et al. (2013) (Figure 7E). Ramified microglia have long thin processes and are thought to be in a surveilling, homeostatic state. In contrast, hypertrophic and amoeboid microglia have larger cell bodies and thicker processes, and are thought to be in an immune activated or phagocytic state (Karperien et al., 2013; Kumar et al., 2016; Anttila et al., 2017; Woodburn et al., 2021). With these criteria, four distinct microglia subtypes were identified and counted: Ramified/MHCII-, Ramified/MHCII+, Hypertrophic/Ameboid/MHCII-, and Hypertrophic/Ameboid/MHCII+. When assessing the total density of microglial phenotype in perilesional gray and sublesional white, there were no significant between group differences in either region of interest (Figures 7F,G, Student’s *t*-test, *p* > 0.05). However, in perilesional gray matter specifically, we found a significant interaction between field and group: in field 1 (closest to the lesion) and field 4 (farthest from the lesion) of perilesional gray, there is a significantly greater density of ramified/MHCII- and total ramified microglia in MSC-EV compared to vehicle group (Figure 7H: field 1, *p* = 0.034; field 4, *p* = 0.044; Figure 7I: field 1, *p* = 0.027; field 4, *p* = 0.017).

When comparing the overall percentages of each microglial phenotype, we found a significant effect of treatment. We found that compared to vehicle monkeys, the ratio of total ramified:hypertrophic/ameboid (Ram: Hyp/Am) microglia were significantly greater in the perilesional gray matter of MSC-EV treated monkeys (Figure 7J, Vehicle = 0.15 Ram: Hyp/Am, MSC-EV = 0.27 Ram: Hyp/Am, *p* = 0.00097). This pattern was also evident in the subset of MHCII+ microglia, which exhibited a significantly higher ratio of Ram: Hyp/Am in MSC-EV compared to vehicle perilesional gray matter (Figure 7K, *p* = 0.041). In the perilesional gray matter of MSC-EV brains, there was a higher percentage of the microglia that are ramified/MHCII- (Figure 7L, Vehicle 9.7%, MSC-EV 15.4%, *p* = 0.038). These between-group differences were only seen in the perilesional gray matter and did not extend to the sublesional white matter. The higher proportion of total ramified microglia, especially the ramified/MHCII- subpopulation, can indicate a more homeostatic/anti-inflammatory micro-environment in perilesional gray matter of MSC-EV relative to vehicle monkeys (Anttila et al., 2017; Woodburn et al., 2021).

We then assessed whether there are longitudinal differences in microglial morphology across recovery, by directly comparing the data from the current cohort at 6-weeks post-lesion (Figure 7L), to our archived data from the 16-week survival cohort from our previous studies (Go et al., 2020) (Figure 7M). The archived dataset consist of aged female monkeys of the same age range (16-26yo) as the current 6-week cohort (Go et al., 2020). Two-way ANOVA (treatment x timepoint) revealed a significant main effect of time on the proportion of Hypertrophic/Ameboid/MHCII- (*p* = 0.0017) and an interactive effect of treatment x timepoint on ramified/MHCII+ microglia (*p* < 0.0001). For both groups the proportion of Hypertrophic/Ameboid/MHCII- was significantly lower in the 16-week (Vehicle 16.0%, MSC-EV 21.1%) compared to the 6-week (Vehicle 66.6%, MSC-EV 63.6%) (Figures 7L,M, Vehicle, *p* = 0.035; MSC-EV, *p* = 0.002). In the MSC-EV group, but not vehicle group, the proportion of ramified/MHCII+ microglia was higher at 16-weeks compared to 6-weeks, increasing from 4.9% at 6-weeks to 34.4% at 16-weeks (Figures 7L,M, *p* = 0.027). These data suggest that in the MSC-EV group the subset of immune activated MHCII+ microglia at 6 weeks may be reverting to a homeostatic, ramified morphological state by 16-weeks post injury.

### 3.8 MSC-EV associated effects on microglial morphology and activation are correlated with plasma inflammatory biomarkers and measures of functional recovery

**Figure 8:**
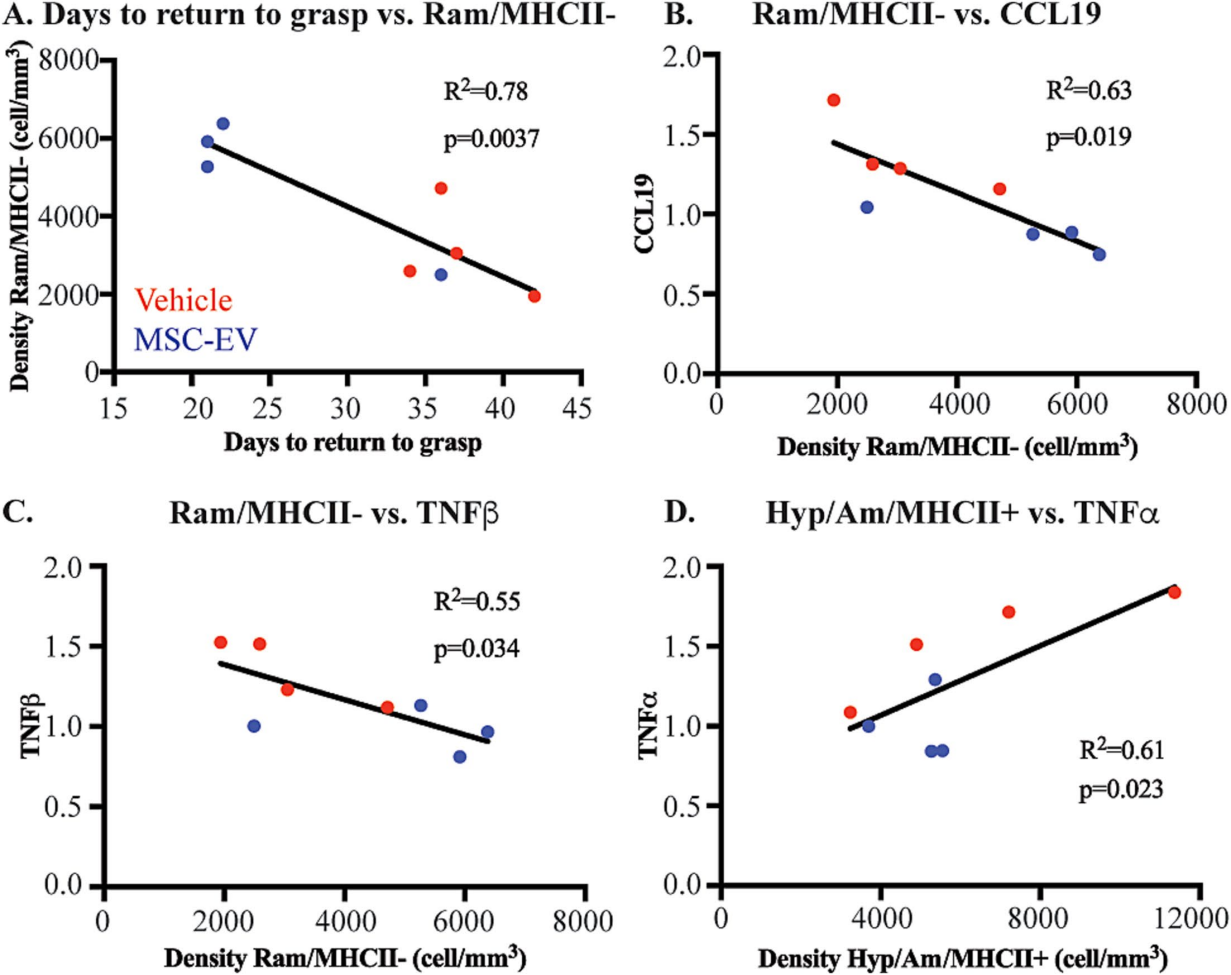
Microglial phenotypes correlate with functional recovery and peripheral inflammatory markers. Scatter plots and linear regression showing significant correlations between: **(A)** Days to return to pre-operative grasp vs. Ramified/MHCII- microglial density **(B)** Ramified/MHCII- microglial density vs. normalized CCL19 plasma concentration at 2 weeks. **(C)** Ramified/MHCII- microglial density vs. normalized TNFβ plasma concentration at 2 weeks. **(D)** Hypertrophic/Ameboid/MHCII+ microglial density vs. normalized TNFα plasma concentration at 2 weeks post-injury. MSC-EV (blue; *n* = 4), Vehicle (red, *n* = 4).

We assessed whether the density of microglia phenotypes correlated with functional outcome measures. We found a significant negative correlation between ramified/MHCII- microglia and the days to return to pre-operative grasp (Figure 8A). In line with our previous study (Go et al., 2020), this data suggests that a more anti-inflammatory, homeostatic environment in the perilesional gray matter is correlated with enhanced recovery of function.

Further, we assessed how these microglial measures of brain neuroinflammation may be related to peripheral changes in plasma inflammatory biomarkers. Interestingly, we found that microglial expression at 6 weeks post injury were significantly correlated with a subset of plasma inflammatory biomarkers at 2 weeks post-injury. Biomarkers in the plasma such as CCL19 (Figure 8B), TNFβ (Figure 8C), CCL11 and TGFα (data not shown) are negatively correlated with the density of homeostatic ramified/MHCII- microglia. These data indicate that higher levels of inflammatory markers in the plasma are correlated with lower density of microglia demonstrating a more homeostatic phenotype. Further, major inflammatory cytokine TNFα (Figure 8D), along with CXCL10 and NT3 (data not shown), were positively correlated with the density of Hypertrophic/Ameboid/MHCII+ microglia. These data show that higher levels of inflammatory plasma proteins are correlated with more pro-inflammatory microglial phenotypes.

## 4 Discussion

Our previous work demonstrated an MSC-EV related enhancement of functional recovery following a selective lesion to the hand representation of the primary motor cortex (Moore et al., 2019), which was associated with reduced microglial inflammation (Go et al., 2020), neuronal damage (Medalla et al., 2020), and increased plasticity and repair (Go et al., 2021; Calderazzo et al., 2022; Zhou et al., 2023) at 16 weeks post-injury. The current study provides evidence of the effects of lesion and MSC-EV treatment on the time course of inflammatory biomarkers in plasma and CSF across recovery and microglial inflammation in brain tissue at a more acute, 6-weeks post-injury timepoint. Specifically, assessments of inflammatory biomarkers using the Olink® PEA demonstrated an MSC-EV related decrease in mainly pro-inflammatory proteins in plasma throughout acute and chronic stages of recovery. The decreased biomarkers are related to pathways associated with peripheral immune cell chemotaxis and the pro-inflammatory response and are correlated with changes in microglial expression in perilesional brain tissue harvested 6-week post-injury. Importantly, these changes are correlated with enhanced functional recovery, suggesting that inflammatory modulation is one key therapeutic target of MSC-EVs that can lead to reduced neural damaged and enhanced repair (Medalla et al., 2020; Go et al., 2021; Calderazzo et al., 2022; Zhou et al., 2023).

### 4.1 MSC-EV treatment modulates peripheral inflammation after cortical injury

The initial acute pro-inflammatory response after cortical injury involves local cytokine and chemokine signaling, reactive oxygen species, and infiltration of peripheral immune cells across the disrupted blood brain barrier (Wang et al., 2007). Pro-inflammatory signaling cascades activate microglia (Gottlieb et al., 2022; Wang et al., 2022) and recruit peripheral immune cells to the brain to contain and clear damage (Kim et al., 2014; Zhu et al., 2022; Lieschke et al., 2019; Simats et al., 2018). This is followed by an anti-inflammatory response that can promote repair of damaged cortical tissue (Jassam et al., 2017; Jin and Yamashita, 2016). Failure to resolve the pro-inflammatory environment can cause further secondary damage and cell death (Postolache et al., 2020). Our current and previous work, as well as studies in rodents, have shown that treatment with MSC-EVs results in an overall suppression of the inflammatory response across the acute and chronic stages, which in turn can mitigate secondary damage and facilitate downstream repair, plasticity and functional recovery (Xin et al., 2013a; Xin et al., 2013b; Xin et al., 2021; Kodali et al., 2023; Wang et al., 2022).

The current study showed that the majority of treatment related DEPs in plasma were found during the acute stage, at 2 weeks post-injury, where both pro- and anti-inflammatory plasma biomarkers are downregulated with MSC-EV treatment. However, the downregulation of anti-inflammatory markers in MSC-EV treated monkeys was specific to the 2-week timepoint but did not persist at 4- and 6-weeks post-injury. As recovery progressed, at 4- and 6-weeks post-injury, MSC-EV treated monkeys showed a sustained downregulation of primarily pro-inflammatory plasma proteins. Functional annotation analyses revealed that these downregulated inflammatory proteins in plasma are implicated in many biological processes associated with functional recovery.

In the early stages of recovery, an acute pro-inflammatory response can be protective after injury, by clearing and containing damage (Anrather and Iadecola, 2016). However, sustained inflammation can promote chronic secondary damage (Schimmel et al., 2017; Kokiko-Cochran and Godbout, 2018) and severe functional impairment (Hou et al., 2021). Specifically, our data showed that the majority of downregulated proteins, mostly found at 2-weeks post-injury, were related to positive modulation of the humoral immune response and the chemotaxis of peripheral immune cells. In addition, our data suggest that MSC-EVs may suppress neurotoxic effects of peripheral immune cells, which have been described to drive secondary inflammation and impair recovery (Zhang Z. et al., 2022). For instance, CCL11, CCL23, and IL-6 have all been shown to attract peripheral immune cells such as macrophages, B-cells, and neutrophils to the site of injury (Zhu et al., 2022; Lieschke et al., 2019; Simats et al., 2018), and all of these proteins are downregulated with MSC-EV treatment. The concurrent downregulation of anti-inflammatory proteins suggests that MSC-EV mediated suppression of acute inflammatory signaling maybe due to an overall lower level of acute damage (Cicchese et al., 2018). This points to a potential neuroprotective mechanism (Kodali et al., 2023; Wang et al., 2022) or facilitation of debris clearance pathways within the very acute stages (Mei et al., 2010; Yin et al., 2023), earlier than 2-weeks post injury. Assessment of biomarkers at even more acute timepoints (e.g., the first days following treatment) would be important to address in future studies.

### 4.2 MSC-EV treatment promotes shift towards homeostatic microglial phenotypes across recovery

As the major immune cells of the brain, microglia play a critical role in recovery after cortical injury. After an insult, microglia undergo a transition from the ramified “homeostatic” surveying state to an “inflammatory” reactive state, characterized by short processes and an enlarged cell body, associated with phagocytic and pro-inflammatory functions (Kumar et al., 2016; Anttila et al., 2017). When damage associated markers are released following injury, microglia enter this immune/inflammatory reactive state, where they can release pro-inflammatory cytokines such as TNFα, interleukins, and reactive oxygen species (Lull and Block, 2010). These reactive microglia proliferate and gather in the damaged areas and can phagocytose debris and damaged cells (Jia et al., 2021). Cytokines released from microglia can attract peripheral immune cells to the lesion, which can further contribute to the pro-inflammatory environment (Berchtold et al., 2020). While clearance of damaged tissue is necessary for recovery, sustained activation of microglia and infiltrating peripheral immune cells can activate apoptotic pathways that further secondary neurodegeneration (Schimmel et al., 2017; Galgano et al., 2017). The role of microglia in recovery from cortical injury and in promoting secondary damage underscores the necessity for a therapy that can target these cells.

In the current study, we found that MSC-EV treatment reduced the expression of inflammatory microglial phenotypes identified based on morphology and MHCII expression. We found that at 6-weeks post injury, MSC-EVs were associated with a higher proportion of ramified microglia, specifically the Ram/MHCII- subtype, which are associated with homeostatic functions (Anttila et al., 2017; Woodburn et al., 2021), in perilesional grey matter. These data suggest that the transition to a less inflammatory environment is occurring by 6-weeks post-injury, leading to the more resolved inflammatory micro-environment seen at the chronic 16-week recovery time point (Go et al., 2020; Zhou et al., 2023). Comparisons of these longitudinal changes in microglial phenotypes across recovery from our current and previous data (Go et al., 2020) revealed that in both treatment groups, there was a transitional decrease in the proportion of inflammatory hypertrophic/amoeboid microglia from 6 to 16-weeks post-injury. However, in the MSC-EV treated group, there was a specific increase in the proportion of ramified, MHCII+ immune stimulated/antigen presenting microglia. These data support the idea that MSC-EV treatment may decrease inflammatory hypertrophic/ameboid MHCII+ microglia via signaling them to shift back to ramified, homeostatic states over time, resulting in more ramified MHCII+ microglia at 16 weeks (Go et al., 2020; Zhou et al., 2023). These data are consistent with rodent *in vivo* and *in vitro* models of injury and neurodegenerative disease showing that MSC-EVs alter the phenotypes of microglia (Hao et al., 2022; Go et al., 2020; Zhou et al., 2023; Mukai et al., 2021) and reduce pro-inflammatory microglial markers (Garcia-Contreras and Thakor, 2021).

Taken together, our current and previous studies in monkeys (Go et al., 2020; Zhou et al., 2023) provide evidence that MSC-EV treatment following cortical injury leads to a decrease in inflammatory microglia starting at 6 weeks post-injury, with a more pronounced shift towards anti-inflammatory phenotypes at 16 weeks post injury. This increase in homeostatic microglia correlated with a more rapid recovery of pre-injury grasp, suggesting a role of this microglial phenotypic shift in functional recovery.

### 4.3 Relationship between peripheral and central inflammatory markers after cortical injury: congruent effect of MSC-EVs on plasma biomarkers and microglia expression

Our data from the current study and in previous work (Go et al., 2020; Zhou et al., 2023) indicate that MSC-EVs modulate both the peripheral and central inflammatory responses after injury, which may underlie enhancements in functional recovery. However, the direct versus indirect targets of MSC-EVs and the relationship between peripheral and central inflammatory responses in our model remain open questions. Levels of CSF inflammatory biomarkers have been shown to predict cortical injury outcome in humans (Naik et al., 2023). However, the current study did not reveal significant effects of MSC-EV treatment on CSF biomarkers. Considering that cytokine expression can peak at 3 days post-injury, and then return to baseline levels with time (Doll et al., 2014; Nayak et al., 2012), it is possible that we are not capturing the earliest injury- and treatment-related changes in CSF biomarkers. The fact that we did not see MSC-EV effects on CSF inflammatory markers, but did observe robust changes in plasma and in brain tissue, underscore the complex relationship between peripheral and central neuro-immune signaling reported in the literature (Zang et al., 2022). Further, understanding the biodistribution of the MSC-EVs would be important to assess in future studies to understand the complex peripheral-central blood–brain relationships.

Our data revealed significant correlations between plasma biomarkers at 2 weeks post-injury and microglial phenotypes at 6-weeks post injury. Specifically, these correlations indicate that higher levels of inflammatory markers in the plasma are correlated with lower density of microglia demonstrating a more homeostatic phenotype. Conversely, positive correlations were found between multiple inflammatory proteins (TNFα, CXCL10, NT3) in plasma 2-weeks post injury and the density of inflammatory (Hyp/Am/MHCII+) microglia subtype in brain tissue. These data suggest that the effects of MSC-EVs on inflammatory signaling early in recovery (2 weeks) can affect the brain microenvironment at later recovery timepoints (6 weeks). To build upon the findings here, direct assessments of plasma and brain extracellular fluids (e.g., through microdialysis) at earlier timepoints across recovery, together with parallel proteomic and transcriptomic profiling of microglia, could further elucidate these neuroimmune signaling mechanisms (Thiollier et al., 2018; Dyhrfort et al., 2019). Future assessments of neuron-derived EVs in plasma at these acute timepoints could further illuminate peripheral-central biomarker interactions that may be important for recovery (Ledreux et al., 2020). Since the current study does not discriminate microglia from invading peripheral macrophages (Zhang Z. et al., 2022; Zhang et al., 2019), further characterization of distinct subpopulations of immune cells at the transcriptomic and proteomic level would also be important (Anttila et al., 2017; Butler et al., 2024). Collectively, these types of parallel multi-modal datasets would be critical to understand the interactions between peripheral and central inflammation. Overall, we have found strong evidence to support the role of MSC-EVs in modulating acute peripheral inflammation, which may lead to a long-term reduction in neuroinflammation, facilitating functional recovery from cortical injury.

## 5 Conclusion

The current study shows that MSC-EV mediated downregulation of inflammatory plasma proteins was associated with the suppression of pro-inflammatory signaling pathways. Further, the MSC-EV effects on plasma biomarkers of inflammation were associated with shifts from pro-inflammatory to anti-inflammatory microglial phenotypes across recovery. This shift in inflammatory brain environment can promote neuronal plasticity and repair at more chronic recovery time points. Importantly, MSC-EV dependent downregulation of peripheral and central inflammatory markers was correlated with a more rapid and enhanced recovery of fine motor function. In summary, the data from both our current and previous studies (Go et al., 2020; Medalla et al., 2020; Go et al., 2021; Calderazzo et al., 2022; Zhou et al., 2023) collectively point to the potential for MSC-EVs to suppress inflammation beginning early and extending into later stages of recovery after cortical injury. Our findings highlight the translatable clinical potential of MSC-EVs as a therapeutic that can target both acute and chronic peripheral and microglial inflammation, to support and enhance recovery following cortical injury.

## Data Availability

The raw data supporting the conclusions of this article will be made available by the authors, without undue reservation.
