# Supplementary material for: Reduction of inflammatory biomarkers underlies extracellular vesicle mediated functional recovery in an aged monkey model of cortical injury
Source: Front Aging Neurosci. 2025 Jul 9;17:1605144. doi: 10.3389/fnagi.2025.1605144 (PMC12283759; doi:10.3389/fnagi.2025.1605144)
Supplement: Supplementary file 2 [file Supplementary_file_1.docx]

Supplementary Materials

# Supplementary Figure 1


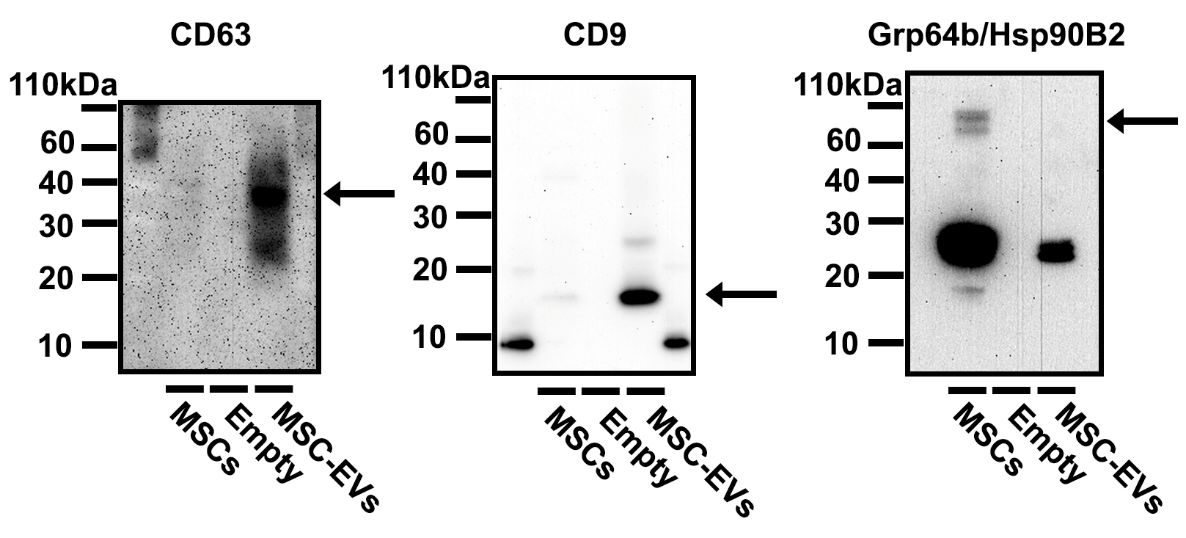


**Supplementary Figure 1. The characterization of MSC-EVs.** Representative full-length Western blot images show that the tetraspanin proteins CD63 (Santa Cruz, sc-5275, RRID:AB_627877) and CD9 (Abcam, ab223052, RRID:AB_2922392) are abundantly present in MSC-EVs. In contrast, the endoplasmic reticulum (ER) chaperone protein Grp64b (also known as Hsp90B2) (Abcam, ab64182, RRID:AB_1141018) is detected only in MSCs, but not in MSC-EVs. These findings meet the criteria outlined in the Minimal Information for Studies of Extracellular Vesicles 2018 (MISEV2018) and MISEV2023 guidelines, confirming that the isolated MSC-EVs have canonical EV markers and exhibit minimal contamination from cellular organelles. Arrows indicate the detected protein bands at the expected molecular weight.

# Supplementary Figure 2


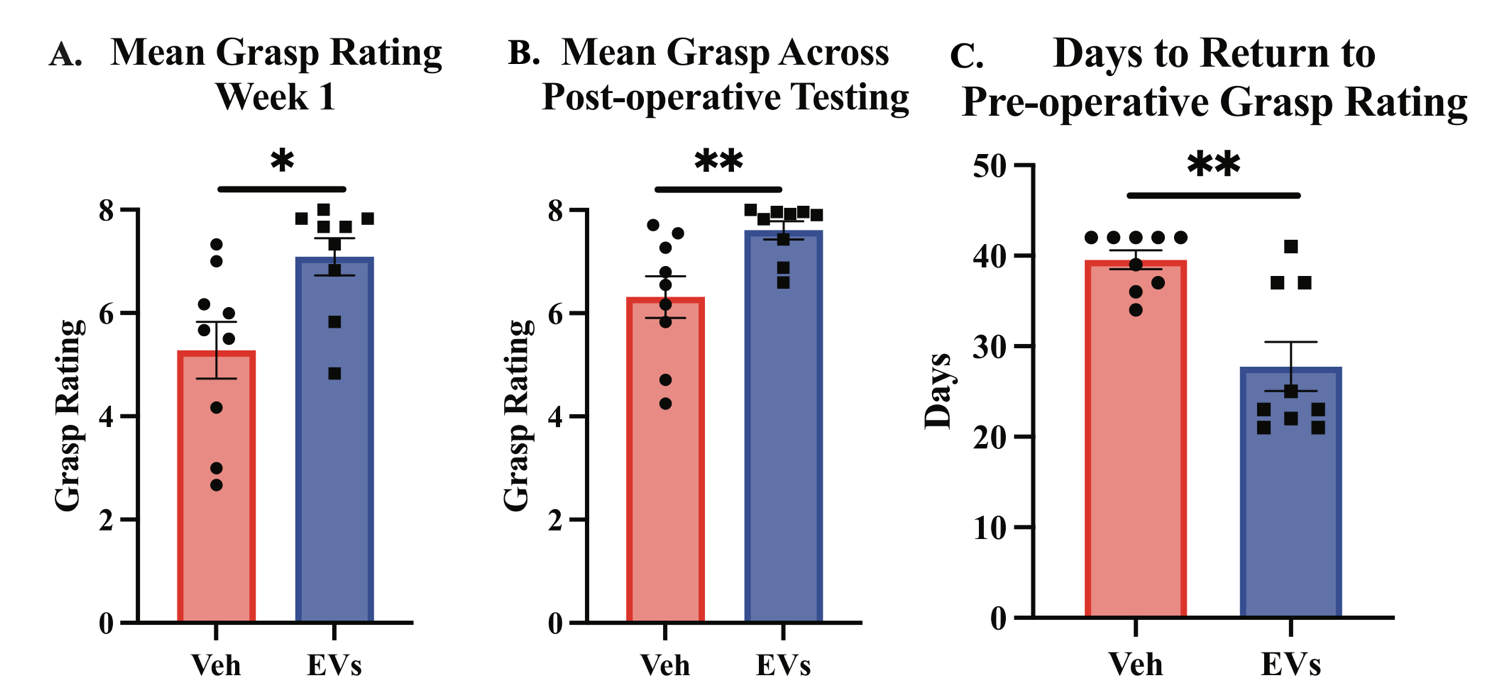


**Supplementary Figure 2.** **Pooled recovery data from both cohorts.** Data set of both the 16 week and 6-week recovery cohorts pooled. **(A.)** The mean grasp rating in the first week of post-operative testing, days 15-21. **(B.)** The mean grasp rating across the first four weeks of post-operative testing (Only the 16-week cohort went beyond these weeks of testing) **(C.)** Days to return to pre-operative grasp. For monkeys that did not reach recovery or plateau within the first 6 weeks, days to return to grasp is recorded as 42 days. Vehicle (red, n=9), MSC-EV (blue, n=9), *= p<0.05, **=p<0.01.

**
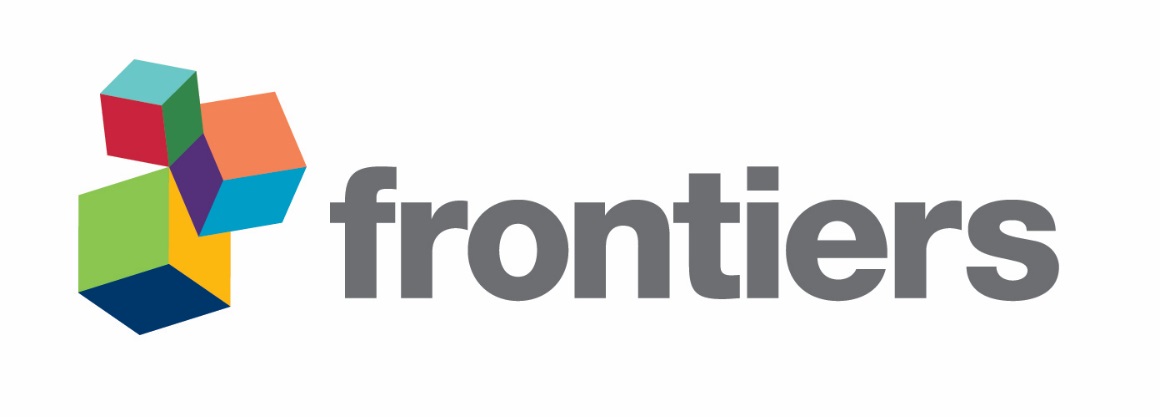
**
